# Upwelling and nutrient dynamics in the Arabian Gulf and sea of Oman

**DOI:** 10.1371/journal.pone.0276260

**Published:** 2022-10-21

**Authors:** Kaltham Abbas Ismail, Maryam R. Al Shehhi

**Affiliations:** Civil Infrastructure and Environmental Engineering, Khalifa University, Abu Dhabi, UAE; Universite Libre de Bruxelles, BELGIUM

## Abstract

This study demonstrates the vertical and horizontal distribution of nutrients and the seasonal response of nutrients to upwelling in the Arabian Gulf and the Sea of Oman. Thus, monthly data on nitrate, phosphate, and silicate are obtained from the World Ocean Atlas 2018 (WOA), as well as estimates of coastal and curl driven upwelling in both regions. The results of the study indicate that the Sea of Oman’s surface and deep waters contained higher concentrations of nutrients than the Arabian Gulf by 80%. In addition, both regions have exhibited a general increase in the vertical distribution of nutrients as the depth increases. Among the aforementioned nutrients, nitrate is found to be a more limiting nutrient for phytoplankton growth than phosphate as the nitrate-to-phosphate ratios (N:P) in surface waters are lower (≤ 4.6:1) than the Redfield ratio (16:1). As for the upwelling, curl-driven upwelling accounts for more than half of the total upwelling in both regions, and both play an important role in nutrient transport. Thus, nutrients are upwelled from the subsurface to the mixed layer at a rate of 50% in the Oman Sea from 140 m to 20 m during the summer and to 40 m during the winter. Similarly, the Arabian Gulf shows 50% transport for nitrates, but 32% for phosphates, from 20 m to 5–10 m. However, due to the abundance of diatoms at the surface of the Arabian Gulf, the surface silicate content is 30% higher than that of the deeper waters.

## 1. Introduction

Photosynthetic organisms need nutrients to produce essential biomolecules such as carbohydrates, proteins, and lipids required for growth and reproduction [1]. The nutrients needed are divided into macronutrients and micronutrients [2,3]. The macronutrients include carbon (C), nitrogen (N), sulfur (S), phosphorus (P), silica (Si), magnesium (Mg), calcium (Ca), and potassium (K), which are consumed in greater quantities compared to the micronutrients [4]. Whereas micronutrients are the metal/metalloid constituents of enzymes that perform biological functions [5] including chlorine (Cl_2_), manganese (Mn), iron (Fe), zinc (Zn), and copper (Cu) [6,7].

These macro and micronutrients exist in the oceans with varying distribution. Among the world’s oceans, the Southern Ocean has the highest amount of macronutrients [8]. Besides, the Arctic Ocean contains significant amounts of micronutrients, such as iron mainly through river runoff, dust and sediments deposited in shallow coastal waters [9]. In addition, a significant portion of the ocean’s net primary production comes from the Indian Ocean (20%) [10]. This high productivity has affected nutrients supply to the coasts along the Indian Ocean and primarily the Arabian Gulf and Sea of Oman, which are the focus of this study. The Arabian Gulf has a pressured marine ecosystem due to the growing population along its coast. More people mean more treated wastewater from residential and industrial areas is discharged into the Gulf, increasing the concentration of nutrients in seawater, causing a phenomenon called eutrophication [11]. For instance, phosphate discharge rate in domestic liquid waste released to the northern part of the Arabian Gulf waters is around 8,294 ton yr^−1^ which is regarded to be high contributing to the total nutrients budget in the region [12]. In addition, the nutrients in the Gulf can come from natural sources as well in which the nutrients of C and N are atmospherically derived elements whereas Ca, Mg, K, and P are minerals derived from rocks and soils [13]. In addition, the intensity of upwelling, advective supply and turbulent mixing could transport the nutrients in horizonal and vertical directions depending on the climate forcing [14].

Yet, little is known about the nutrients and their sources in the Arabian Gulf in which few studies have been conducted. As an example, Kuwait waters has been studied to measure nutrients levels at six sites in Kuwait Bay and compared with points selected in the Arabian Gulf. Kuwait Bay exhibited higher mean concentrations for inorganic nutrients than the Arabian Gulf with values of 1.5–1.6 μg L^−1^, 0.6–0.7 μg L^−1^, and 33.5 μg L^−1^ for NO_x_ (nitrite plus nitrate), DIP (dissolved inorganic phosphorous), and silica respectively [15]. In addition, 27 locations were sampled in deep and offshore stations midway between the Qatari and Iranian coasts and the maximum nitrate concentrations were less than 4–5 μM [16]. Another recent study by [17] investigated the nutrients distribution at 20 stations between 25°N and 27°N across the Arabian Gulf and the Sea of Oman indicating insignificant silicate differences between the two regions (2.73–2.96 μM) while exhibiting high concentrations of phosphate (0.74–1.10 μM). This is resulted from northeast monsoon’s upwelling with the N:P ratio (10:1) lower than the Redfield ratio 16:1 which describes the average composition of phytoplankton biomass. Redfield ratio is a widely accepted stoichiometric reference for nutrient limitation of planktonic production [18]. A lower N:P ratio than Redfield ratio therefore indicates nitrogen is the main limiting nutrient in the region.

Given the few studies mentioned above, the nutrient of the Arabian Gulf is still overlooked as many resources that describe chemical processes across the Gulf basin are outdated and few studies have been conducted on this topic. Thus, we have chosen to study the nutrient distribution in the Arabian Gulf owing to these reasons and some other interesting qualities of the region, including: 1) seasonal changes in river run over time, especially in the northern area [19], 2) frequent sandstorms throughout the year especially between May and July (e.g. an average of 8 sandstorms occur in Kuwait each year) [20], 3) high salinity on average of 40–41 psu [21], and 4) frequent algal bloom outbreaks (e.g. >3 events per year) [22].

Therefore, the spatial and temporal variability of the nutrients in the form of nitrate (NO_3_), phosphate (PO_4_), and silicate (SiO_4_), are analyzed herein for the Arabian Gulf and the Sea of Oman. Moreover, the seasonal upwelling’s effects on the distribution of nutrients are explained. Therefore, the Ekman method is used together with the sea surface temperature (SST) upwelling index [23] to identify the upwelling regions and their cooling effect. In particular, the derived vertical velocities of curl-driven upwelling and coastal upwelling based on the Ekman transport components are used to quantify the upwelling caused by wind stress and wind stress curl. As upwelling could provide significant nutrients to coastal marine ecosystems [24–29], the nutrient profiles of the entire region over the upwelling regions (Arabian Gulf and Sea of Oman) are also explored.

## 2. Data and methods

### 2.1 Datasets

**Fig 1:**
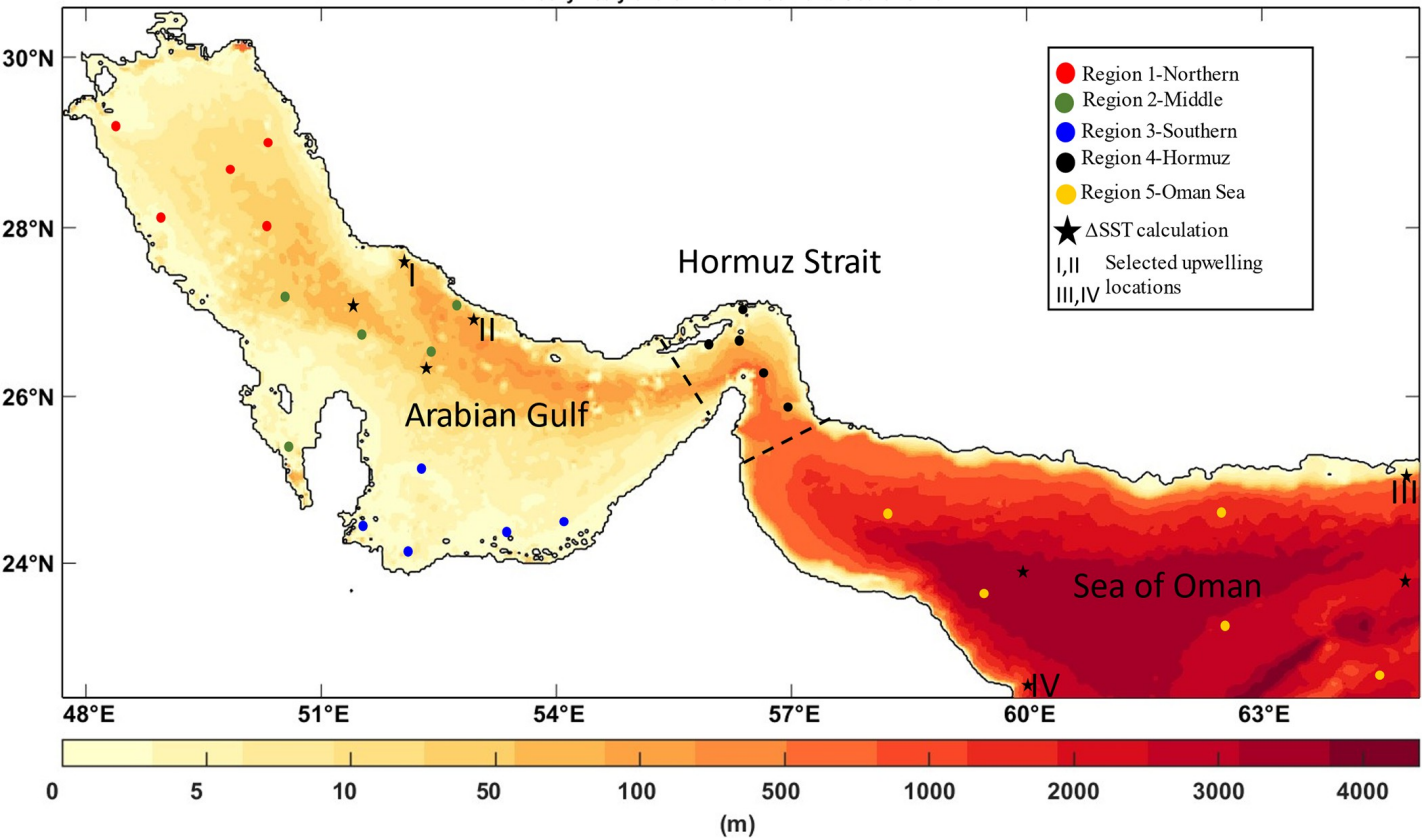
Bathymetry of the Arabian Gulf and the Sea of Oman map. The filled circles are locations where the inorganic nutrient’s vertical profile is plotted, red: Region 1-Northern; green: Region 2-Middle; blue: Region 3-Southern; black: Region 4-Hormuz Strait; orange: Region 5-Sea of Oman. The black stars represent places where the upwelling index is calculated using a simple thermal difference of the ocean and coastal SST: *UI*_*SST*_ = *SST*_*coast*_−*SST*_*ocean*_, while the Greek numbers (I, II,III,IV) are regions of upwelling where inorganic nutrients are analysed.

The domain selected for this analysis comprised of the Arabian Gulf and Sea of Oman which is divided into three sub-regions: Arabian Gulf, Hormuz (transition area), and Sea of Oman to study the surface and vertical distribution of nutrients, see Fig 1. Records of monthly macronutrients, nitrate (NO_3_), phosphate (PO_4_), and micronutrient silicate (SiO_4_), have been obtained from the global World Ocean Atlas (WOA) 2018: https://www.ncei.noaa.gov/access/world-ocean-atlas-2018) [30]. The WOA data are extensively used as initial and boundary conditions as well as for model validation in many biogeochemical modelling studies [31–40] assuring its reliability and accuracy in many regions. These data consist of a set of objectively analyzed climatological fields with a spatial resolution of 1 degree at standard depths.

In order to perform Ekman transport estimations (section 2.2.2), monthly climatology data in the form of 10 m zonal and meridional wind components *U*_10_ and *V*_10_ have been obtained from the European Center for Medium-Range Weather Forecasts Interim Reanalysis (ERA-Interim): https://www.ecmwf.int/en/forecasts/datasets/reanalysis-datasets/era-interim) for the year 2018 (ECMWF, 2018). ERA-Interim is a product of global atmospheric reanalysis that optimally combines model data and observations from a variety of sources to produce a consistent, global, and optimized estimates of numerous atmospheric and oceanic parameters. The inputs *U*_10_ and *V*_10_ consist of 3D grids with latitude, longitude, and time in each dimension. The spatial resolution of the wind data is 0.75 × 0.75 degrees, and it has been resampled as nutrients data. For the estimation of SST upwelling index, VIIRS monthly Level 3 SST data have been acquired from NASA’s Ocean Color Database: https://oceandata.sci.gsfc.nasa.gov with a resolution of 4 km for the Arabian Gulf and Sea of Oman.

### 2.2 Methods

As a first step towards examining the spatial variability of the aforementioned nutrients, seasonal nutrient maps, as well as profiles of nutrients across the Arabian Gulf and the Sea of Oman, are generated. The Redfield ratios are calculated afterwards to determine which nutrient is limiting in each region. Furthermore, the Ekman transport method is utilized to measure the depth of the Ekman layer in the Arabian Gulf and Sea of Oman and identify the regions of curl driven upwellings and coastal upwellings. Coastal upwellings have also been examined based on the SST method. The details are shown below.

#### 2.2.1 Mapping the seasonal surface and vertical nutrients

The monthly nutrients data (nitrate, phosphate and silicate) have been resampled and interpolated into a grid size of 360 × 180 × 43 (longitude, latitude, depth) for the Arabian Gulf and Sea of Oman domain with latitudes: [22.3738 − 30.5765°N] and longitudes: [47.6979 − 65.0104°E]. In order to analyze seasonal variations in nutrients during summer and winter, the monthly data for December, January, and February were averaged as winter and June, July, and August as summer. Using these averaged data, surface nutrient maps for winter and summer are generated. Similarly, nutrient seasonal profiles have also been extracted but only for five sub-regions, including the northern Arabian Gulf, the center of the Arabian Gulf, the southern Arabian Gulf, the Strait of Hormuz, and the Sea of Oman. The bathymetry of the Arabian Gulf does not exceed 100 m, while that of the Sea of Oman could exceed 3 km. Therefore, in total, 15 locations have been selected in the Arabian Gulf, and 10 in the Strait of Hormuz and Sea of Oman as shown in Fig 1.

#### 2.2.2 Redfield ratio

In order to determine the limiting nutrients in the Arabian Gulf and the Sea of Oman, the ratios of mean seasonal (i.e. summer and winter) nitrate (NO_3_) to phosphate (PO_4_) ratios for both surface and depth averaged concentrations are calculated (Table 1). The ratios (N:P) are then compared with the Redfield ratio (16:1), with a lower ratio representing nitrogen limitation and a higher ratio representing phosphorus limitation.

**Table 1:** Seasonal variability of nitrate, phosphate, and silicate (μM) of the averaged surface and averaged depth waters in the Arabian Gulf, Hormuz, and the Sea of Oman extracted from the monthly climatology of the World Ocean Atlas Data (WOA) for the year 2018. The WOA data are climatological data collected from the periods 1900–2017. The calculation of the average depth waters is based on the average of several depths. Summer (June, July, August); winter (December, January, February).

**Surface**	**Area**	**The Arabian Gulf (AG)**								**The Transition Area (Hormuz)**								**The Sea of Oman (SO)**							
	Parameter (*μM)*	Summer				Winter				Summer				Winter				Summer				Winter			
		**Max**	**Min**	**Mean**	**STD**	**Max**	**Min**	**Mean**	**STD**	**Max**	**Min**	**Mean**	**STD**	**Max**	**Min**	**Mean**	**STD**	**Max**	**Min**	**Mean**	**STD**	**Max**	**Min**	**Mean**	**STD**
	**Nitrate**	0.16	0.09	**0.14**	0.06	0.29	0.05	**0.19**	0.035	0.77	0.005	**0.2**	0.091	3.25	0.24	**0.57**	0.42	1.02	0.01	**0.51**	0	3.25	0.24	**0.87**	0.57
	**Phosphate**	0.43	0.13	**0.21**	0.04	0.50	0.13	**0.24**	0.40	0.27	0.16	**0.23**	0.034	0.55	0.17	**0.51**	0.08	0.55	0.16	**0.39**	0.04	0.55	0.17	**0.51**	0.11
	**Silicate**	3	0.24	**1**	0.55	2.18	0.58	**1.12**	0.48	2.18	0.58	**0.9**	0.31	3.50	0.67	**1.32**	0.72	4.08	0.58	**2.38**	0.59	5.38	0.67	**3.46**	1
**Bottom**																									
	**Nitrate**	0.18	0.003	**0.16**	0.06	0.29	0.05	**0.16**	0.035	9	0.005	**0.32**	0.091	5.8	0.21	**0.28**	0.42	11.4	0.01	**2.4**	0	6.64	0.0006	**1.24**	0.57
	**Phosphate**	1.74	0.13	**0.17**	0.04	1.70	0.13	**0.17**	0.40	2.82	0.13	**0.32**	0.034	0.77	0.005	**0.20**	0.08	2.89	0.13	**0.97**	0.04	3	0.13	**0.95**	0.11
	**Silicate**	11.9	0.24	**0.44**	0.55	2.85	0.24	**1**	0.48	61.5	0.24	**1.52**	0.31	3.5	0.67	**1.63**	0.72	62.85	0.24	**10.6**	0.59	63.8	0.24	**10.6**	1

#### 2.2.3 Ekman method

For the purpose of studying the distribution of nutrients in relation to upwelling, advection and mixing of water, we calculated the monthly Ekman transports to calculate vertical velocities associated with open sea upwelling from the curl of the wind, vertical velocity of coastal upwelling and total vertical velocity in addition to the Ekman depth as shown below.

- Ekman transports

First, Ekman transport components (*U*_*E*_, *V*_*E*_) [m^3^ s^−1^ m^−1^] at each grid point (0.75 degrees) is calculated based on the wind data obtained from ECMWF datasets by applying Eqs 1 and 2:

$$U_{E}=\frac{\tau_{y}}{\rho_{w}f}$$ (1)

$$V_{E}=-\frac{\tau_{x}}{\rho_{w}f}$$ (2)

Where *U*_*E*_ and *V*_*E*_ are the zonal and meridional Ekman transports, *ρ*_w_ = 1025 kg m^−3^ is seawater density, f = 2Ωsinθ is the Coriolis parameter where Ω = 7.292 × 10^−5^ rad s^−1^ is the Earth’s angular velocity, and *θ* indicates the latitude. The wind speed (*U*_10_, *V*_10_) is automatically computed and converted into wind stress (*τ*: N m^−2^) with the subscripts x and y indicating zonal (along shore wind stress) (*τ*_x_) and meridional (*τ*_y_) components using Eqs 3 and 4,

$$\tau_{x}=\rho_{a}C_{d}({U_{10}}^{2}+{V_{10}}^{2}{)}^{1/2}$$ (3)

$$\tau_{y}=\rho_{a}C_{d}({U_{10}}^{2}+{V_{10}}^{2}{)}^{1/2}$$ (4)

where *ρ*_a_ = 1.22 kg m^−3^ is the density of air and C_d_ = 1.3 × 10^−3^ is the drag coefficient (dimensionless).

- Ekman layer depth

Considering that Ekman currents decrease exponentially with depth. The thickness of the layer is arbitrary and the velocity at the depth (*D*_*E*_) [m] that is opposite to velocity at the surface is considered as the Ekman thickness or Ekman depth [41]. The Ekman layer depth is computed by Eq 5.

$$D_{E}=\frac{7.6}{\sqrt{sin|\varphi |}}(U_{10},V_{10})$$ (5)

where φ is the latitude.

- Vertical velocity of curl-driven upwelling

The curl-driven upwelling (w_curl_) at the base of the Ekman layer is calculated from the divergence of the Ekman transport as shown in Eq 6.

$$\left(\frac{\partial U_{E}}{\partial x}+\frac{\partial V_{E}}{\partial y}\right)=\nabla \cdot U_{E}=-(w_{\mathrm{surface}}-w_{curl})=w_{curl}$$ (6)

where U_E_ is the horizontal Ekman transport and ∇ is the horizontal divergence operator. Ekman vertical velocities approach zero at the sea surface (w_surface_ = 0). Hence,

$$w_{curl}=\nabla \cdot U_{E}=\frac{\partial }{\partial x}\left(\frac{\tau_{y}}{\rho_{w}f}\right)-\frac{\partial }{\partial y}\left(\frac{\tau_{x}}{\rho_{w}f}\right)=\left(\nabla \times (\frac{\vec{\tau }}{\rho_{w}f})\right)\cdot \vec{k}\approx \frac{1}{\rho_{w}f}(\nabla \times \vec{\tau })\cdot \vec{k}$$ (7)

where $\vec{\tau }$ is the vector wind stress and $\vec{k}$ refers to the unit vector in the vertical direction. The wind stress derivatives at each grid point (0.75 degrees) are obtained. Positive wind stress curl produces Ekman suction (upwelling) and negative curl produces Ekman Pumping (downwelling).

- Vertical velocity of coastal upwelling

Using the offshore Ekman transport associated with the predominant alongshore wind stress (m^3^ s^−1^ per meter of coast) calculated above we can determine the vertical velocity of the coastal upwelling (*w*_*coast*_) [m s^−1^] as shown in Eq 8,

$$w_{coast}=\frac{U_{E}}{R_{d}}$$ (8)

where *R*_*d*_ = 1 ×10^3^ km is the Rossby radius of deformation. This average value of *R*_*d*_ is determined by applying Eq 9 to calculate the average R_d_ acquired at each grid point for the entire region.


$$R_{d}=\frac{{gD}^{0.5}}{f}$$ (9)

Where g is the gravitational acceleration and D is the water depth. The total vertical velocity (*w*_*T*_) of coastal upwelling and curl-driven upwelling can therefore be obtained by adding the vertical velocity of both processes as shown in Eq 10,

$$w_{T}=w_{coast}=w_{curl}$$ (10)

The positive values of *w* represent upwelling (i.e. upward velocity) and negative values represent downwelling (downward velocity) [41,42].

#### 2.2.4 SST upwelling index

An additional upwelling index, derived from the difference between the coastal and offshore SST (ΔSST), is used to confirm the upwelling results obtained by the Ekman method. Eq 11 shows the equation used to calculate the resultant SST upwelling index (*UI*_*SST*_).


$${UI}_{SST}={SST}_{coast}-{SST}_{ocean}$$ (11)

Where *SST*_*ocean*_ is that which is 0.5 degrees from the coasts of the Arabian Gulf and 2 degrees from the coasts of the Sea of Oman. Whereas *SST*_*coast*_ is SST observed right along the coast. In the case of a negative *UI*_*SST*_, the coastal waters are cooler than the open ocean, indicating upwelling, while a positive value showing the opposite, indicating no upwelling.

## 3. Results and discussion

### 3.1 The spatial variability of surface nutrients

**Fig 2:**
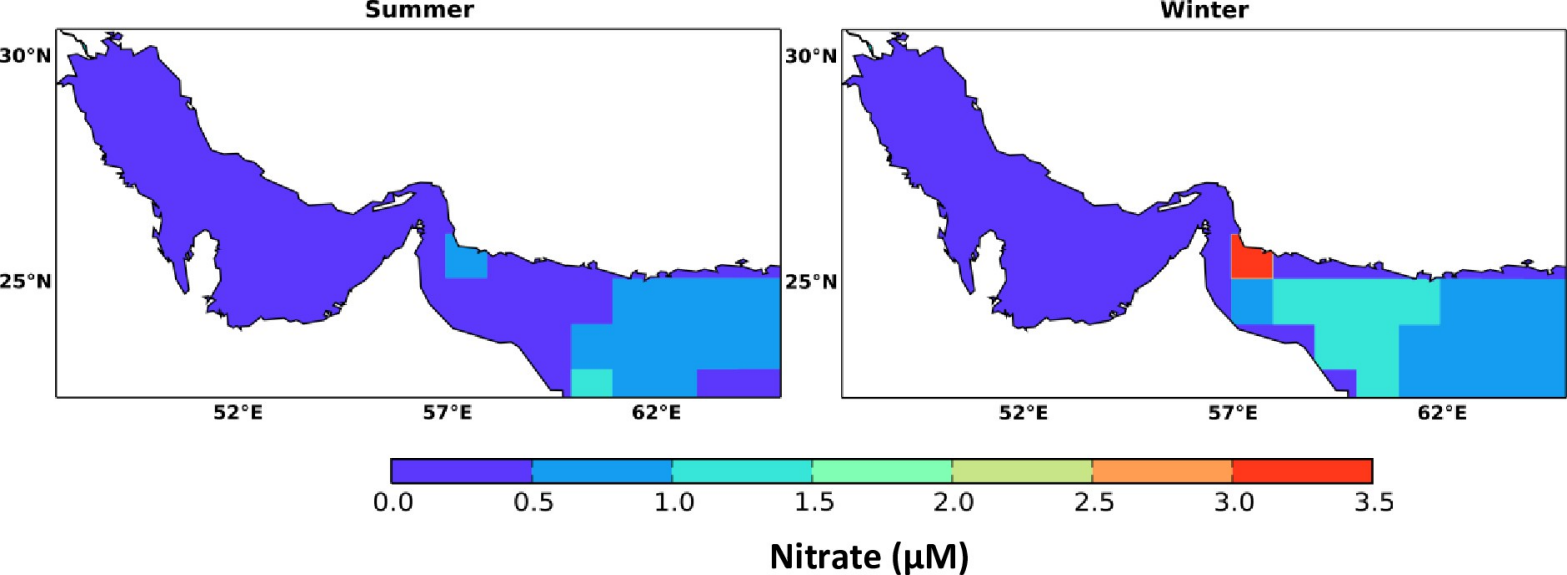
Seasonal spatial distribution of ocean surface nitrate in the Arabian Gulf and Sea of Oman calculated from the monthly climatology of the World Ocean Atlas data (WOA) for the year 2018. The WOA data are climatological data collected from the periods 1900–2017.

Based on the WOA nutrients data extracted for the Gulf and Sea of Oman, the surface waters of the Arabian Gulf are found to exhibit unexpectedly much lower concentrations of nitrate ranging between 0 to 0.16 μM compared to concentrations of 0.005 − 3.25 μM and 0.01 − 3.25 μM in the Strait of Hormuz and the Sea of Oman, respectively. However, high surface nitrate concentrations (maximum of 3.25 μM) have been detected near the Iranian waters (Fig 2) in the Arabian Gulf. The concentration of nutrients in Arabian Gulf waters is less than that of the Sea of Oman owing to its shallowness and well mixed water column. In addition, the pelagic biogeochemistry in the Arabian Gulf is highly impacted by the sedimentary processes allowing direct exchanges between the surface with the materials in the seafloor [19]. This is observed also in the Atlantis II data (1977) where the maximum nitrate was found to be around 3.9 μM [43]. The Atlantis II data has been collected during Atlantis II cruises in winter in the north-western Arabian Sea and the Sea of Oman [40], hence it is used for supporting the observations here. Based on the seasonal (summer: June, July, August; winter: December, January, February) spatial distribution, surface nitrate exhibits higher levels in winter than summer by 36% in the Gulf. Similarly, in the Sea of Oman, the concentrations of surface nitrate are higher by 71% in winter than in summer, as indicated in Fig 2 and Table 1. However, the nitrate distribution has not shown significant variation in a monthly basis, so nitrate monthly maps are not included in the results.

**Fig 3:**
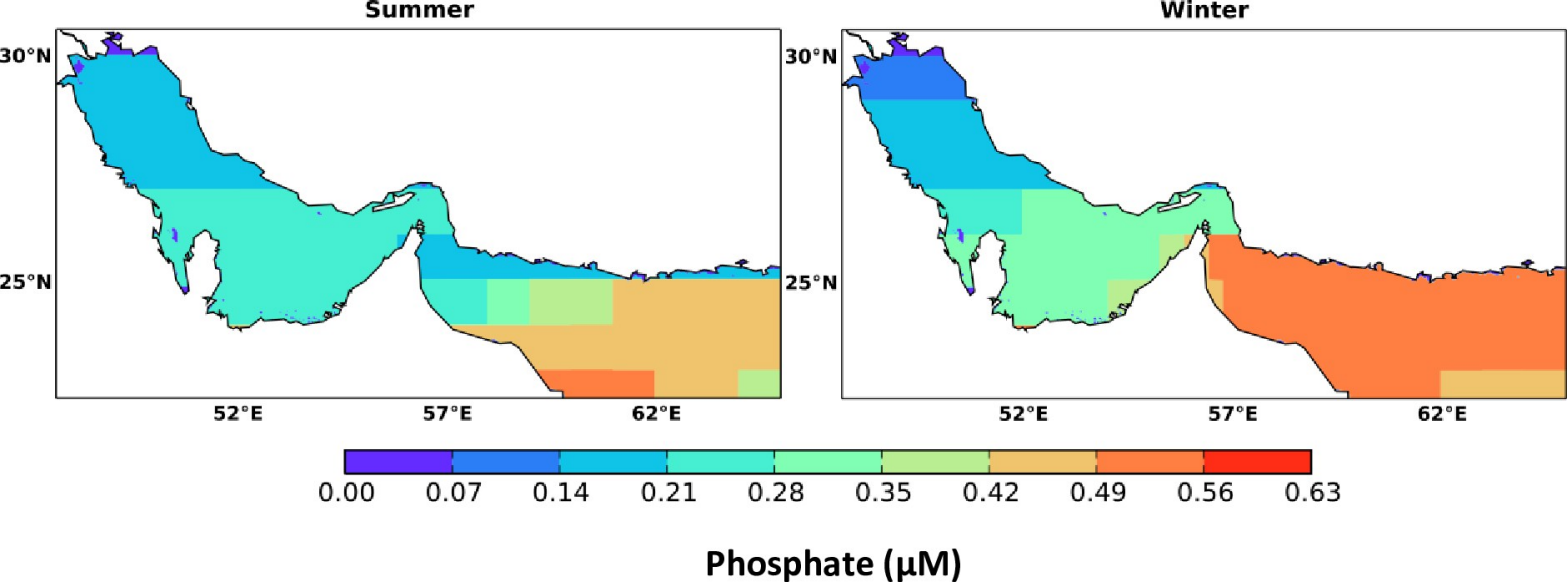
Seasonal spatial distribution of marine surface phosphate in the Arabian Gulf and Sea of Oman extracted from the monthly climatology of the World Ocean Atlas data (WOA) for the year 2018. The WOA data are climatological data collected from the periods 1900–2017.

Unlike nitrate, phosphate revealed a pronounced seasonal and monthly spatial variability in both the Gulf and the Sea of Oman. The average surface concentrations of phosphate in the Gulf, Hormuz Strait, and the Sea of Oman during summer are 0.21, 0.23 and 0.39 μM and during winter are 0.24, 0.51 and 0.51 μM, respectively. Phosphate levels are shown to be higher during summer (0.14 − 0.21 μM) than in winter (0.07 − 0.14 μM) at the northern part of the Gulf whereas the southern part exhibits slightly lower concentrations during summer (0.21 − 0.28 μM) than in winter (≤ 0.28 μM) at the Gulf wide basin. Compared to Atlantis II data (1977), phosphate levels in the northern part of the Gulf are observed to be in the range of 0.1− 0.15 μM. In the southern part of the Gulf, higher concentrations of phosphate is shown especially along the coastal line of the United Arab Emirates (UAE) with values around 0.45 μM in both seasons which is consistent with a study conducted in the southern Arabian Gulf waters during winter by [44] showing that phosphate concentrations could reach up to 0.84 μM. Compared to the Gulf, phosphate contents are found to be higher in the Sea of Oman. Obviously, the water of Sea of Oman has shown high seasonal variations of phosphate with higher concentrations in winter (mainly above 0.49 μM) than in summer (mainly between 0.14 and 0.49 μM)—see Fig 3. Thus, waters rich in phosphate flow from the Sea of Oman into the north entering the Gulf through the Strait of Hormuz enriching the Gulf through physical processes (e.g. mixing, advection, and Ekman transport) and biogeochemical processes (e.g. oxidation of labile dissolved and particulate organic matter) [45,46]. On a monthly basis, phosphate has shown a significant variability during different months of the year (Fig A1 in S1 Appendix). For example, in summer months (June, July, August) the northern part of the Gulf has shown higher levels of phosphate (0.15 − 0.35 μM) compared to the rest of months whilst the southern part of the Gulf has shown insignificant differences in almost all months with values ranging from 0.15 to 0.45 μM. Nevertheless, the southeastern part of the Gulf (close to the Strait of Hormuz) has revealed relatively higher values of phosphate which can reach up to 0.45 μM. In regard to Sea of Oman, phosphate levels can reach up to 0.75 μM particularly in August (max 0.9) and February (max 1 μM). Whereas, in February, the concentration is 1 μM along the Iranian side which is most probably got advected from the Sea of Oman alike the Atlantic II data [43].

**Fig 4:**
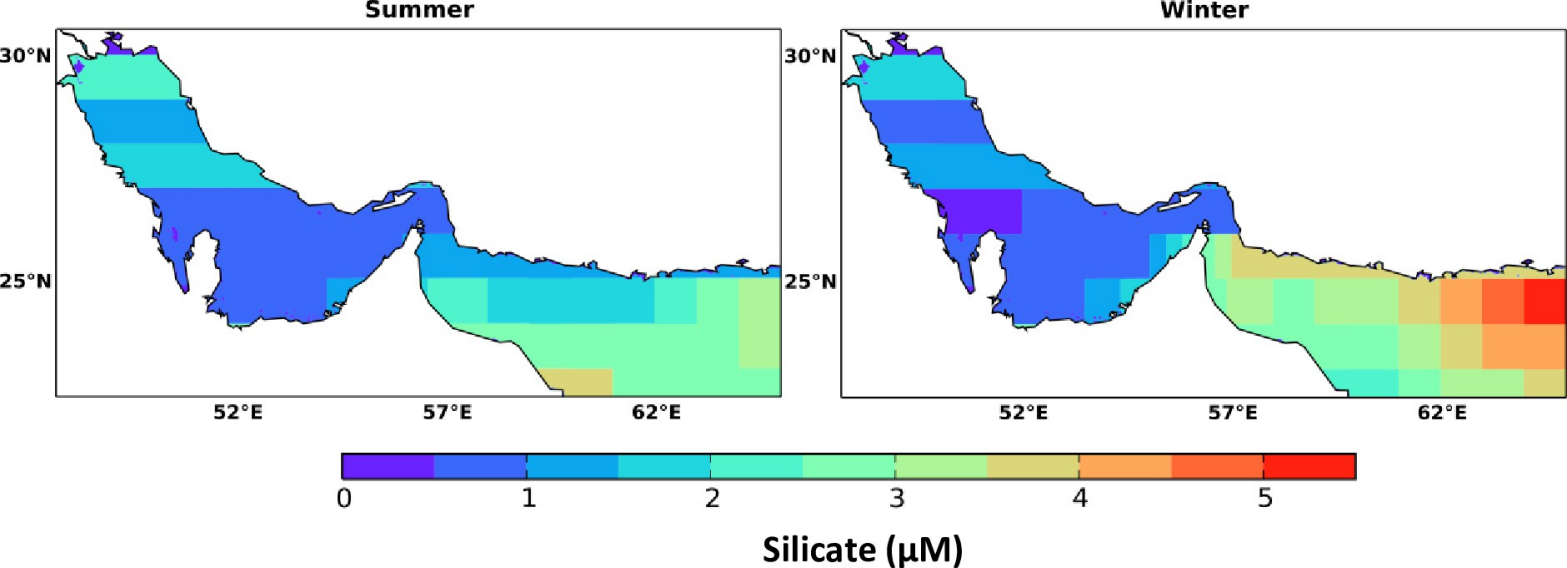
Seasonal spatial distribution of marine surface silicate in the Arabian Gulf and Sea of Oman extracted from the monthly climatology of the World Ocean Atlas data (WOA) for the year 2018. The WOA data are climatological data collected from the periods 1900–2017.

Similar to phosphate, surface silicate revealed a clear seasonal variation in the Arabian Gulf and Sea of Oman but with higher variability in the Sea of Oman during the winter season. The mean values of silicate in the Arabian Gulf, Strait of Hormuz, and the Sea of Oman during summer are 1, 0.9, and 2.38 μM and during winter are 1.12, 1.32 and 3.46 μM, respectively. The spatial concentration of silicate is found to be higher at the northern part of the Arabian Gulf in summer (mainly between 1 and 2.5 μM) compared to that in winter (not exceeding 2 μM) as shown in Fig 4. While the southern part of the Arabian Gulf has illustrated insignificant seasonal differences with values ranging between 0 and 1.5 μM except at the coasts of UAE waters showing higher silicate levels of 1 − 2.5 μM during summer and 1 − 3 μM during winter. These ranges are consistent with concentrations of silicate reported by [44] in the southern part of the Arabian Gulf (2.14–6.26 μM). Compared to the Arabian Gulf, silicate has shown higher concentrations in the Sea of Oman especially in its southern region where the ranges of silicate are found to be 1 − 4 μM during summer and 2 − 5 μM during winter. On a monthly basis, silicate monthly variability is prominent in the Sea of Oman (max > 9 μM in September) whereas the Arabian Gulf has not shown significant variations (Fig A2 in S1 Appendix). However, the southern part of Arabian Gulf has shown a clear depletion of silicate in April, May, and June. The high depletion of silicate is also shown at the Sea of Oman in May. The same tendency is also shown in the data of Atlantis II cruise (February 1977) indicating that the inflowing waters from the Sea of Oman have a negligible amount of silicate [43]. This could be indicated by the maximum depth (125 m) in which the water is entrained into surface layer where the concentration of silicate is low [47]. However, at a localized scale silicate could have reached up to 5.9 μM as was the case along the coastal waters of Kuwait in 1998 [48], however, this is not seen in the data presented here. Table 1 shows the mean, maximum, minimum values and standard deviation of the nutrients in the Arabian Gulf and Sea of Oman.

As for the nutrients’ limitations in the Arabian Gulf and Sea of Oman, it is found that the mean surface N:P ratio for the Arabian Gulf is 0.7:1 during summer and 0.79:1 during winter. Similarly, low N:P ratios are found in the Hormuz and Sea of Oman waters with values of 0.9:1 and 1.3:1 during summer and 1.1:1 and 1.7:1 during winter, respectively. Based on the comparison between these values and the Redfield ratio, it can be demonstrated that three regions show nitrate to be the limiting nutrient. These low N:P ratios have been also observed in the Arabian Gulf and Sea of Oman with values of 2.2:1 and 2.7:1 [44].

### 3.2 The vertical variability of nutrients

**Fig 5:**
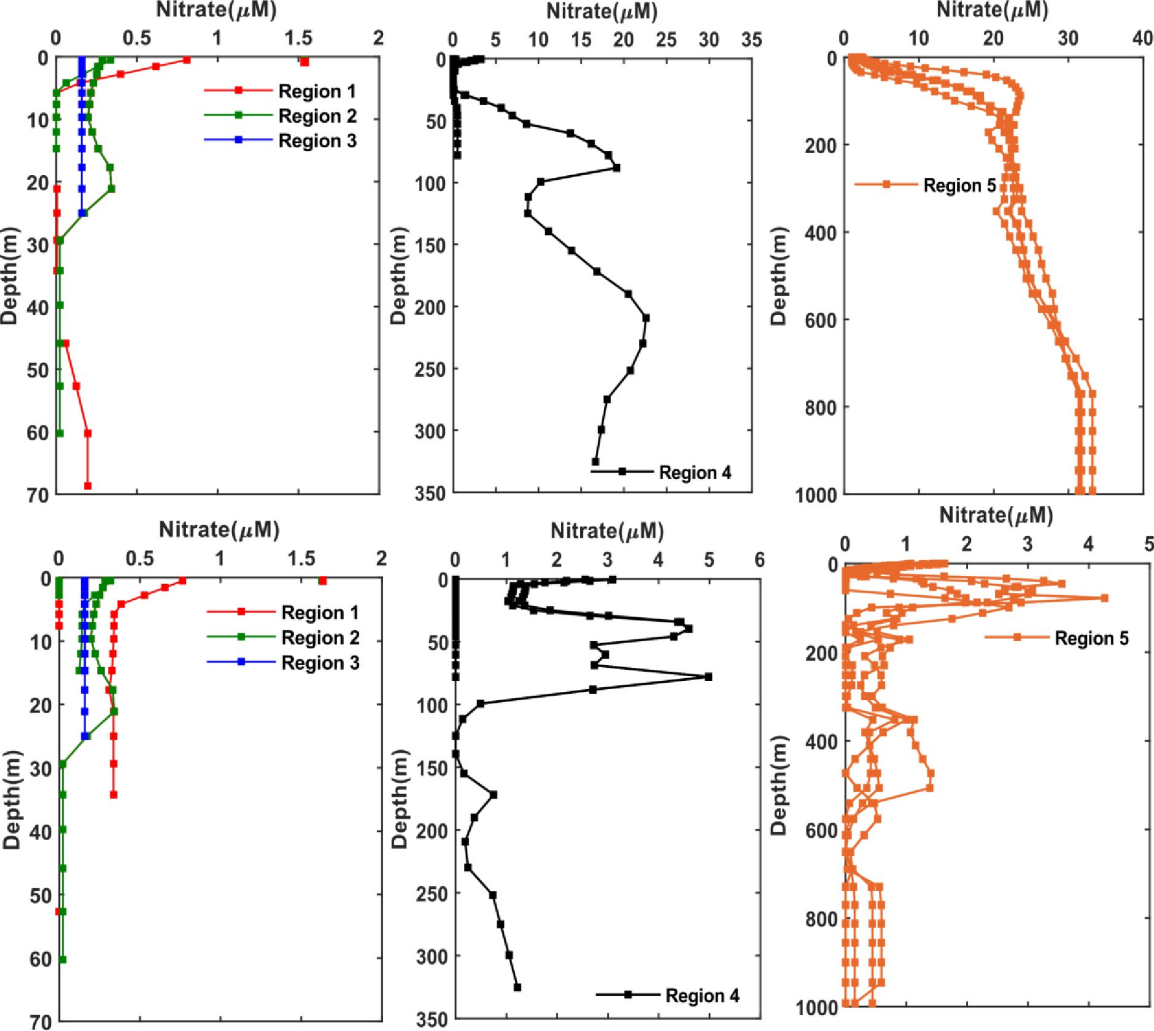
Seasonal nitrate profiles at different regions divided over the Arabian Gulf and Sea of Oman (as per Fig 1) extracted from the monthly climatology of the World Ocean Atlas data (WOA) for the year 2018. The WOA data are climatological data collected from the periods 1900–2017. Top: Summer season (June, July, August); bottom: Winter season (December, January, February). Left: Arabian Gulf, middle: Strait of Hormuz, right: Sea of Oman. The scale used for winter and summer in the Strait of Hormuz and Sea of Oman are different due to the large difference in the concentrations in both seasons and this allow to see the variations better at depth.

The variability of the vertical nutrient profiles is evaluated here for the five sub-regions listed in the methods section, as illustrated in Fig 1. Overall, nitrate shows slight increase with depth in the Arabian Gulf waters (Fig 5) where the highest concentration of nitrate in the Arabian Gulf is found at the northern region of the Arabian Gulf (region 1) nearby Kuwait waters with a value ~1.52 μM during both seasons. Similarly, region 2 has shown slight vertical variability for nitrate with highest concentrations (0.3 μM) at depth of 20 m during both seasons. However, in the southern Gulf (region 3), nitrate concentrations have demonstrated low vertical variability during both seasons, with an average value of 0.2 M throughout the whole water column, possibly due to well mixed waters. This is in contrast to the previous reported values of nitrates in the southern Arabian Gulf region that have shown higher concentrations at localized stations such as: i) 10 μM was observed along the UAE coasts at 20 m depth [49] and ii) summer and winter values of 4.9 μM (depth: 75 m) and 1 μM (depth: 55 m) reported in Qatari waters [16]. Nitrate concentrations have sharply increased in the eastern part of the Strait of Hormuz (region 4) reaching a maximum value of 20 μM at depth of 210 m during summer. The concentration of nitrate has also increased gradually reaching the highest value of 34 μM (depth ≥ 1000 m) in the Sea of Oman waters during summer. These results are consistent with the Atlantis II data [43] where the maximum concentration of nitrate was around to be 39 μM at depth 1444 m in the Sea of Oman. The increase of nutrients in bottom waters may have caused by several factors including organic compounds oxidation, shell dissolution and release from sediments at the bottom waters [50] whereas in surface waters nutrients are used up to near depletion by phytoplankton. In contrast, during winter, the concentrations of nitrate in the Sea of Oman and Strait of Hormuz have declined to below 1 μM (depth ≥ 1000 m) as shown in Fig 5.

**Fig 6:**
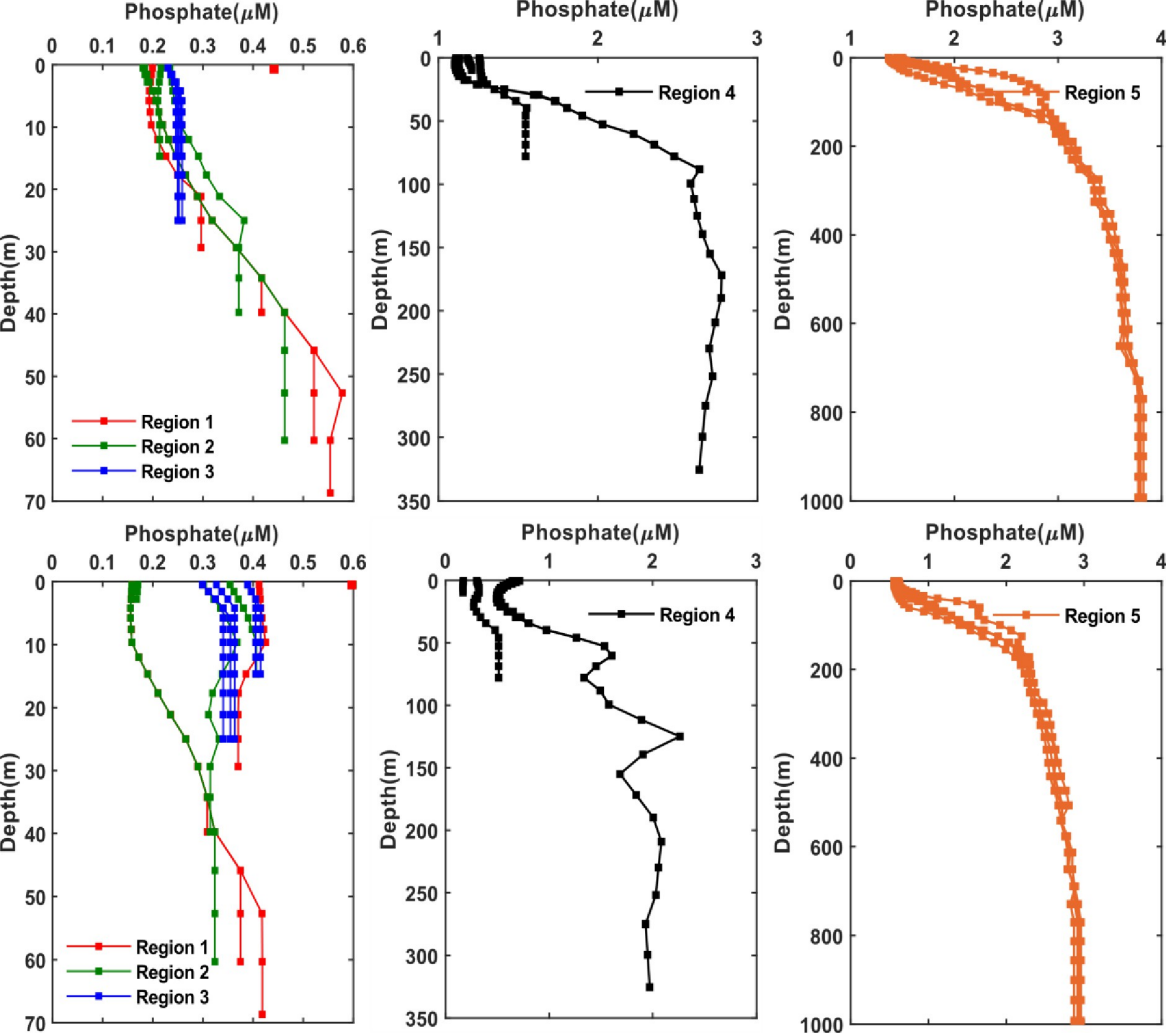
Seasonal phosphate profiles at different regions divided over the Arabian Gulf and Sea of Oman (as per Fig 1) extracted from the monthly climatology of the World Ocean Atlas data (WOA) for the year 2018. The WOA data are climatological data collected from the periods 1900–2017. Top: Summer season (June, July, August); bottom: Winter season (December, January, February). Left: Arabian Gulf, middle: Strait of Hormuz, right: Sea of Oman.

**Fig 7:**
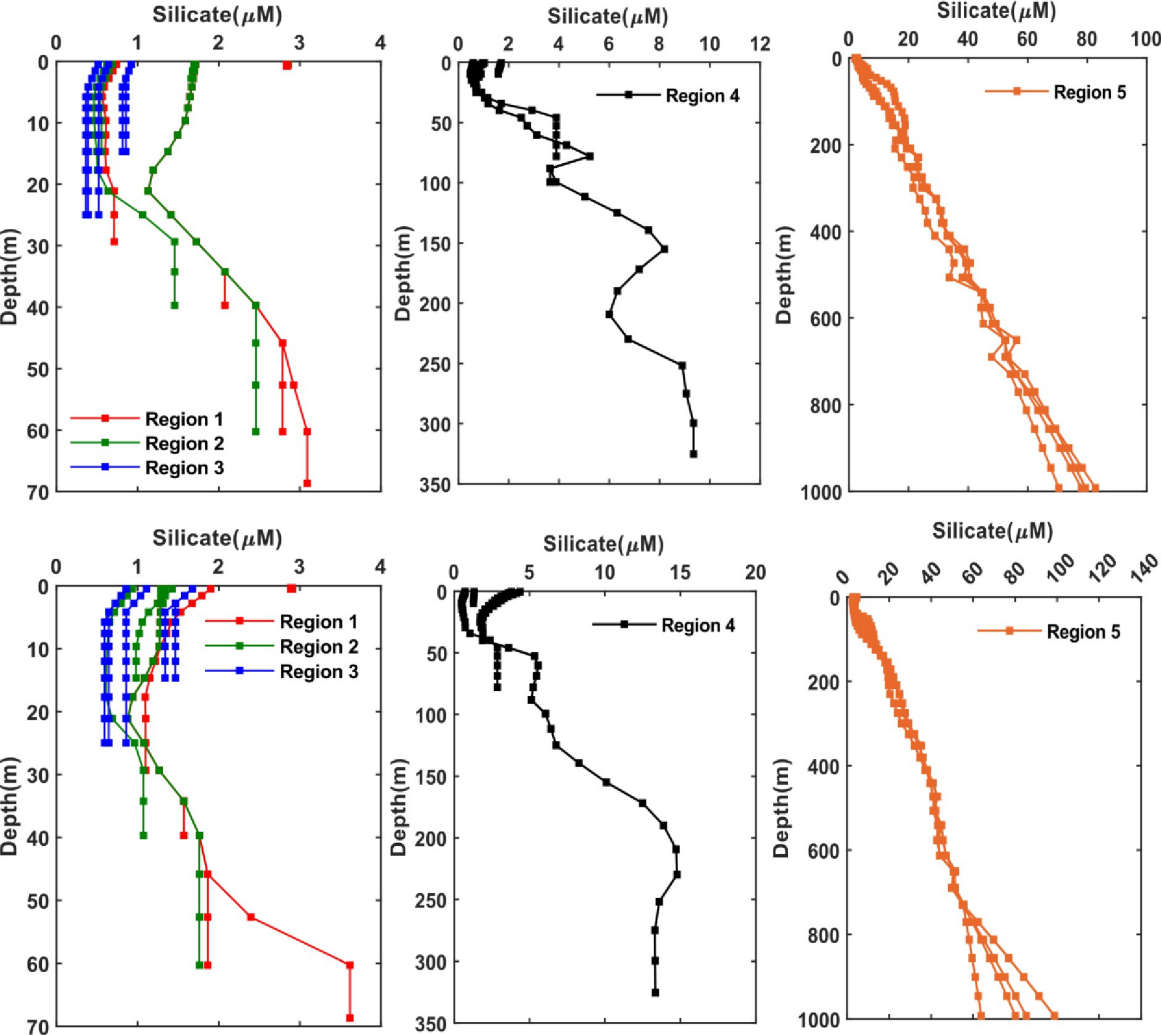
Seasonal silicate profiles at different regions divided over the Arabian Gulf and Sea of Oman (as per Fig 1) extracted from the monthly climatology of the World Ocean Atlas data (WOA) for the year 2018. The WOA data are climatological data collected from the periods 1900–2017. Top: Summer season (June, July, August); bottom: Winter season (December, January, February). Left: Arabian Gulf, middle: Strait of Hormuz, right: Sea of Oman. The scale used for winter and summer in the Strait of Hormuz and Sea of Oman are different due to the large difference in the concentrations in both seasons and this allow to see the variations better at depth.

As for the phosphate, a pronounced increase of phosphate is shown in the bottom waters for all the five regions in both seasons with a slight increase in winter (Fig 6). So, generally the present data have shown maximum levels of phosphate: (> 0.6 μM at depth 2m), (> 2 μM at depth > 75 m), and (3 μM at depth ≥ 1000 m) in the Arabian Gulf, Strait of Hormuz and Sea of Oman during winter. The highest concentration of phosphate (> 0.6 μM) in the Arabian Gulf can be seen in the northern part (region 1) along Kuwaiti waters during winter. However, in the southern part of the Arabian Gulf (region 3) the distribution of phosphate is almost uniform with depth, mainly 0.3 μM in both seasons which is consistent with an earlier study reported the nitrate concentration during winter in the Qatar waters [16]. In the Sea of Oman, phosphate has shown a maximum value of 3 μM (depth ≥ 1000 m) during winter that is matching the value of 2 μM (depth >300 m) reported by [44] as well as [43] with a maximum of 3.8 μM at depth 1154 m. Likewise, silicate has shown an increase with depth in all five regions and within a higher extent in the bottom waters of Strait of Hormuz and Sea of Oman which tend to be higher in winter than summer (Fig 7). The highest concentrations of silicate in the Arabian Gulf are observed in the: i) northern part (region 1) with a value of around 3 μM in both seasons at depth of 2 m, ii) the Strait of Hormuz with concentration of 9 μM at depth ≥ 250 m during summer and 15 μM at depth ≥ 225 m during winter and iii) the observations reported by [51] have indicated sufficient silicate (mean of 5.9 μM) in the coastal waters of Kuwait and can reach up to 5 μM [48]. These silicate concentrations are also consistent with those reported by [49] where the highest concentration was around 12 μM in the southern region of the Arabian Gulf along the coastal line of UAE during winter. However, silicate is found to be concentrated in the bottom water (depth > 1000 m) of the Sea of Oman in both seasons with concentrations reaching up to 120 μM (depth > 1000) in winter and close to 100 μM (depth > 1000 m) in summer.

As for the nutrients’ limitations based on the depth averaged profiles, the N:P ratios show values of 0.9:1, 1:1 and 2.5:1 during summer and 0.9:1, 1.4:1 and 1.3:1 during winter in the Arabian Gulf, Hormuz and Sea of Oman. These ratios are found to be significantly lower than the Redfield ratio (16:1). This is consistent with previous studies of [43,44,49]. However, this is contrary to the standard notion that silicate constitutes the main limiting nutrient for diatoms in the Arabian Gulf. This suggests that nitrate is more essential than phosphate as a limiting nutrient for the phytoplankton growth and the denitrification effect is more pronounced than the nitrogen fixation effect in both the Arabian Gulf and Sea of Oman.

### 3.3 Description of vertical water transports

- Ekman transport and Ekman layer Depth

**Fig 8:**
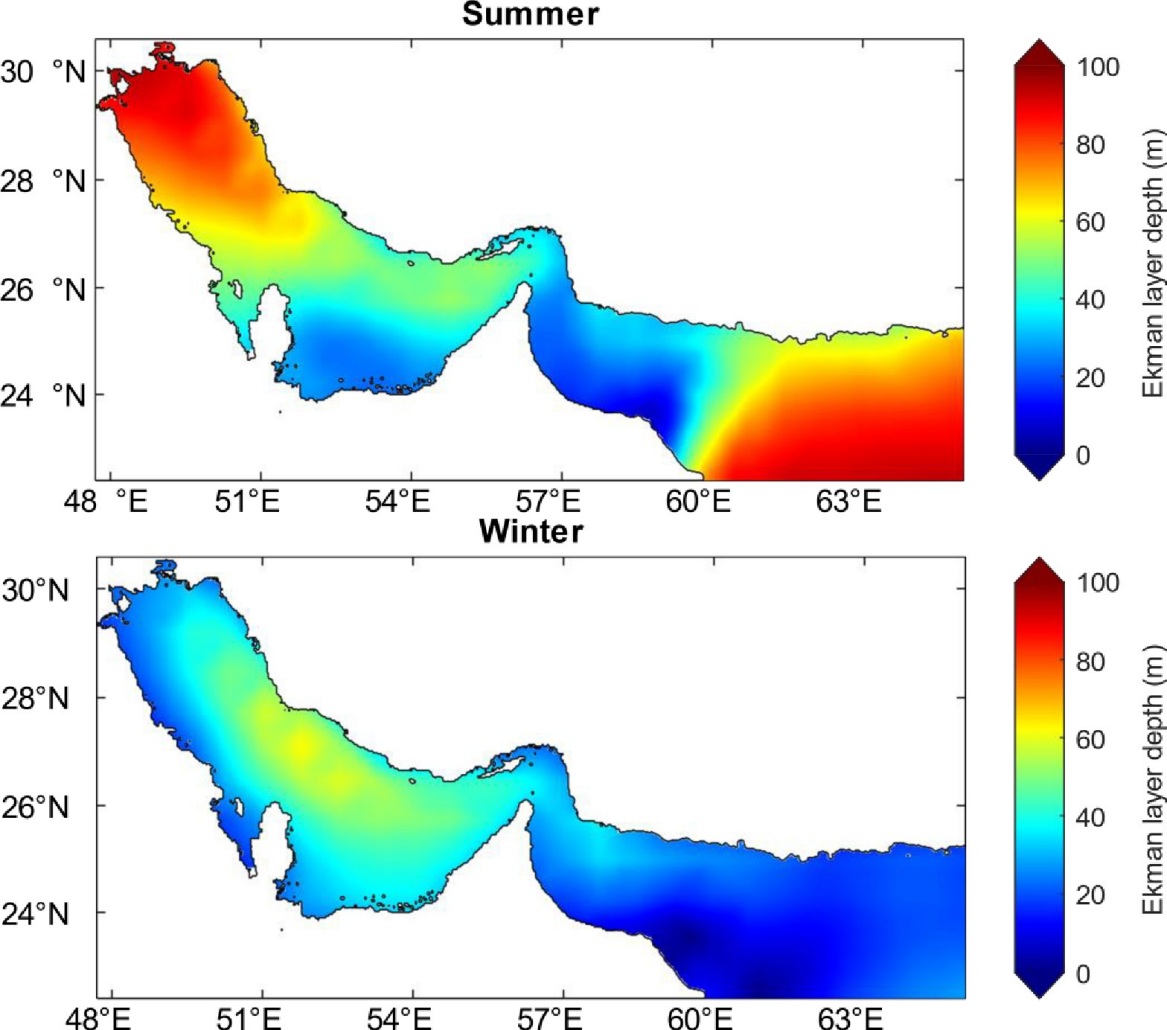
Seasonal Ekman layer depth (m) over the Arabian Gulf and Sea of Oman calculated from the wind speed which is extracted from the ECMWF (ERA5) datasets from the monthly climatology of the year 2018. ERA5 **Climatologies have been calculated over the long-term average period of 1981–2010.** Top: Summer season (June, July, August); bottom: Winter season (December, January, February).

In order to identify the upwelling regions and understand the vertical water transports in the Arabian Gulf and Sea of Oman, Ekman transport has been obtained first for the whole region as described in the methods section. We have found that significant intensity of transport been occurred during the summer months of Jun, July, August, September and the winter month of January as shown in Fig A3 in S1 Appendix. Ekman transport is found to be stronger in the Sea of Oman compared to the Arabian Gulf during the whole year. For example, the offshore transport is the strongest in Sea of Oman (southeast of Oman) during July reaching up to 2.1 m^3^ s^−1^ m^−1^ while in the Arabian Gulf offshore transport doesn’t exceed 1 m^3^ s^−1^ m^-1^ during its peak period (June). During the months between Jun and August, Ekman transport increases from 0.04 to 0.2 m^3^ s^−1^ m^−1^ in the Arabian Gulf and from 1.4 to 2.1 m^3^ s^−1^ m^−1^ in the Sea of Oman. However, it shows a decrease during September (0.02 m^3^ s^−1^ m^−1^) in the Arabian Gulf in contrary to an increase in Sea of Oman (1.4 m^3^ s^−1^ m^−1^). Ekman transport is found to be oriented westward and southward in the Arabian Gulf while in the Sea of Oman they are directed eastward and southward. Ekman transport is also found to be perpendicular to the coastline in the offshore direction of the northern Arabian Gulf (along Iran coast) causing upwelling while it is in the on-shore direction causing downwelling in the western Arabian Gulf (along Saudi coasts). The strong Ekman transport at the northern Arabian Gulf during Jun, July and August has caused the Ekman layer depth to deepen to more than 70 m during June and August and reaching up to 90 m during July (Fig A4 in S1 Appendix). Similarly, Ekman layer depth in Sea of Oman during is found to be deep exceeding 80 m during June and August and reaching up to 100 m during July. However, during September the Ekman layer depth shows low values ranging between 40 and 60 m in the Arabian Gulf and between 60 and 90 m in the Sea of Oman. As for the seasonal variability of Ekman layer depth, it has an average value of 60 m in the Arabian Gulf and Sea of Oman during summer while its average values are 40 m in the Arabian Gulf and 20 m in the Sea of Oman during winter as shown in Fig 8. This variability is explained by the strong wind blowing during summer and weaker wind during winter especially over the sea of Oman (< 5 m s^−1^) [52].

- Upwelling regions and associated cooling effect

**Fig 9:**
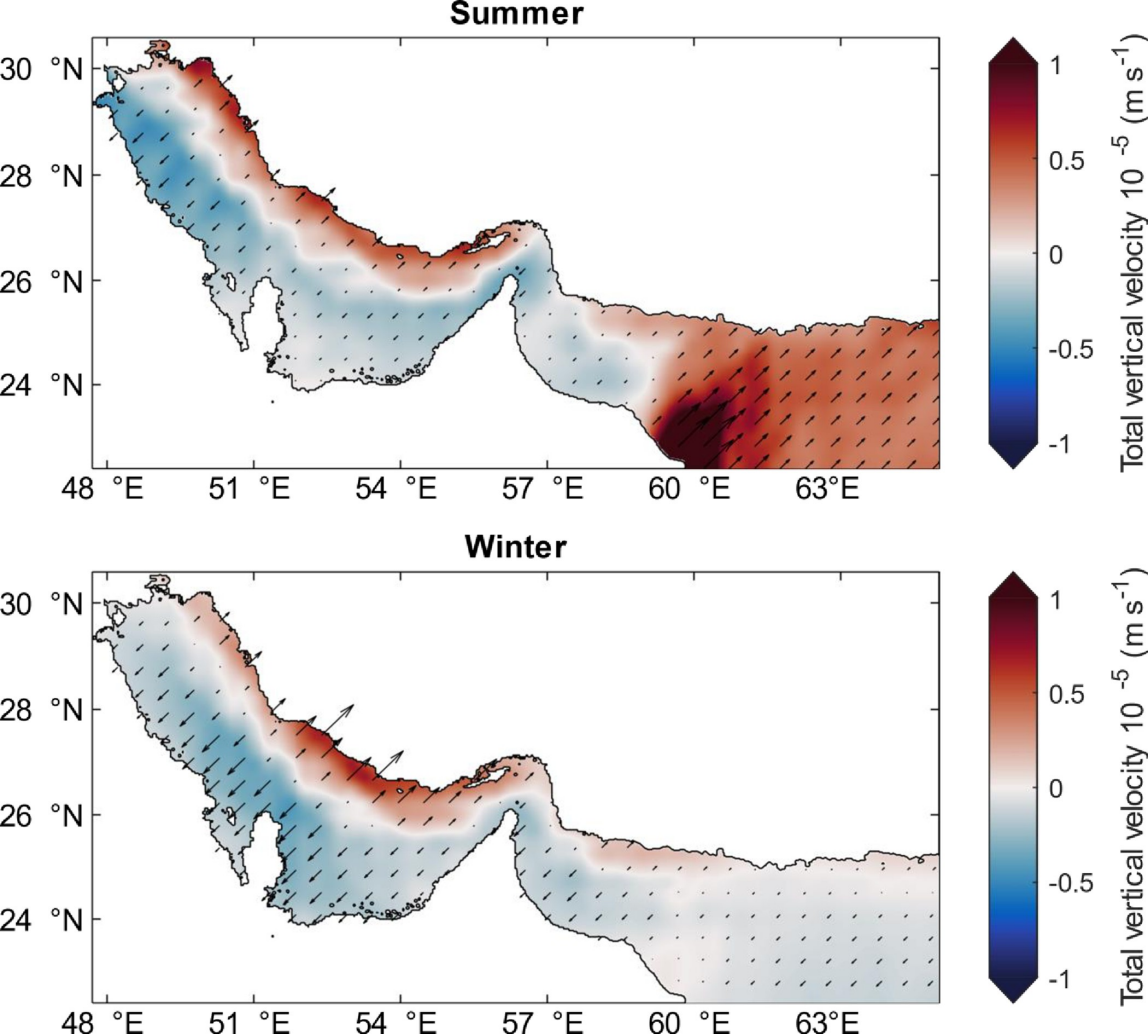
Seasonal total vertical velocity due to alongshore wind stress (coastal upwelling) and wind-curl (open sea upwelling) over the Arabian Gulf and Sea of Oman calculated from the monthly climatology of wind speed parameter which is extracted from the ECMWF (ERA5) datasets for the year 2018. ERA5 Climatologies have been calculated over the long-term average period of 1981–2010. The vectors on the map show the direction of the velocity which is upward for a positive vertical velocity and downward for a negative vertical velocity. Top: Summer season (June, July, August); bottom: Winter season (December, January, February).

**Fig 10:**
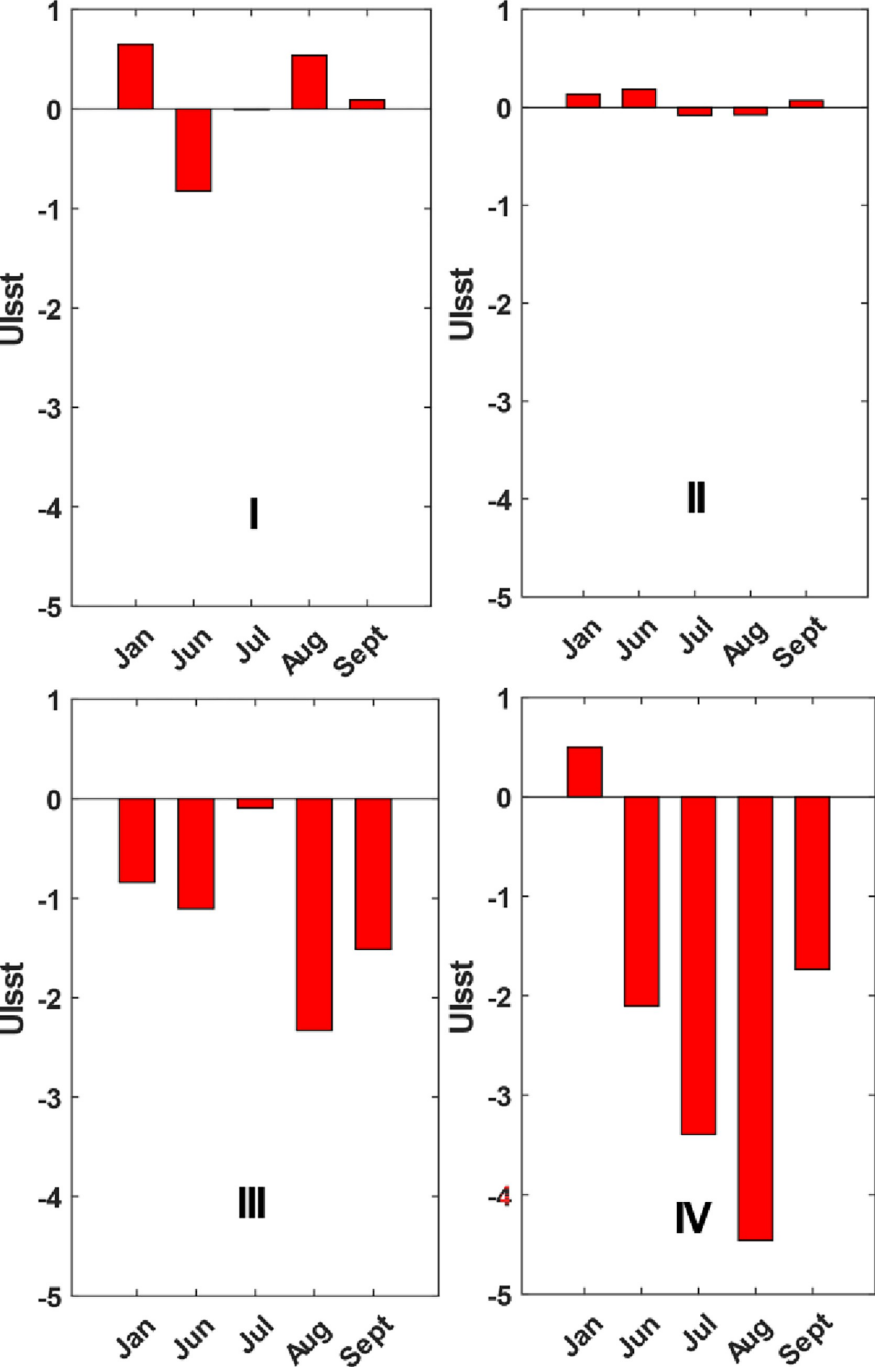
Sea surface temperature (SST) upwelling index (UI_SST_) (unit: ˚C) for the months (January, June, July, August, September) in the Arabian Gulf (I, II) and Sea of Oman (III, IV). SST data is extracted from the SNPP-*VIIRS* monthly Level 3 Sea Surface Temperature (SST) dataset.

Based on the Ekman transport, a total of four upwelling regions have been identified to be occurring during summer in the Arabian Gulf and Sea of Oman where the total vertical velocities exceed 0.5 m s^-1^ (Fig 9). These regions show curl driven upwelling to be dominant compared to the coastal upwelling. Two of these regions are located at the northern Arabian Gulf along Iran coasts (regions I and II), eastern Sea of Oman (region III) and southern Sea of Oman along Oman coasts (region IV) as shown in Fig 1. The upwelling at these regions is a result of strong offshore Ekman transport (> 0.45 m^3^ s^−1^ m^−1^) and high average Ekman layer depth of 60 m during summer as mentioned earlier. In particular, strongest upwelling occurs during June in regions I and II (0.9 x 10^−5^ m s^-1^) and during July in region IV (2.3 x 10^−5^ m s^-1^). This latter also experiences strong upwelling during September (1.6 x 10^−5^ m s^-1^), see Fig A5 in S1 Appendix due to Ekman offshore transport and Ekman depth as shown in Fig A3 and A4 in S1 Appendix and Table 2. During summer, the aforementioned upwelling conditions cause cooling effect at region I during June, region II during July, regions III and IV during August confirmed by the maximum *UI*_*SST*_ values approaching − 0.8, − 0.08, − 2.3 and − 4.5°C, respectively as shown in Fig 10. These negative values of *UI*_*SST*_ indicate that warmer waters at the surface are replaced by cooler water from the bottom which may enrich the surface water with nutrients. This is particularly more significant in the Sea of Oman upwelling regions III and IV compared to the Arabian Gulf upwelling regions I and II. However, during winter weaker upwelling (1.1 × 10^−5^ m s^−1^) occurs compared to summer at regions I and II due to weak offshore transport at these regions. Therefore, no cooling effect has been observed during winter where the maximum *UI*_*SST*_ is 0.6°C in region I and 0.1°C in region II.

**Table 2:** Mean SST upwelling index, Ekman layer depth, vertical velocity of curl-driven upwelling, vertical velocity of coastal upwelling, total vertical velocity, and nutrients concentrations extracted at *a* depth: ~20m in both I and II *and* depth: ~140m at III and IV at the upwelling regions during the summer season (June, July, August). Ekman layer depth and vertical velocity are derived from the monthly climatology of wind datasets which are extracted from the ECMWF(ERA5) datasets for the year 2018. ERA5 Climatologies have been calculated over the long-term average period of 1981–2010. Whereas nutrient’s data are downloaded from the monthly climatology of the WOA data for the year 2018. The WOA data are climatological data collected from the periods 1900–2017.

Upwelling region	UI_SST_ (°C)	Ekman layer depth (m)	w_curl_ (10^−5^ m s^-1^)	w_coast_ (10^−5^ m s^-1^)	Total vertical velocity (10^−5^ m s^-1^)	Nitrate (μM)	Phosphate (μM)	Silicate (μM)	N:P ratio
I	0.38	55	0.60	-0.025	0.57	0.15	0.18	0.57	0.8:1
II	-0.21	45	0.47	-0.0072	0.46	0.16	0.17	0.38	0.9:1
III	-0.58	66	0.40	0.06	0.46	3.4	0.90	5.85	3.8:1
IV	-1.27	67	1.20	0.1	1.30	5.3	1.83	18.2	2.9:1

### 3.4 Effect of upwelling on nutrients transport

After identifying the major upwelling regions in the Arabian Gulf and Sea of Oman, the regional effect of upwelling at these regions on the nutrient distribution is investigated in this section during both the summer and winter seasons. Hence, the analysis herein focuses on the water column in addition to the euphotic zone, which receives nutrients through vertical mixing and coastal upwelling from the thermocline [53,54]. The thermocline is at depth of 10 − 20 m the Arabian Gulf and 100 − 350 m in the Sea of Oman [55]. During the summer season, high concentrations of nutrients between depths of 100 and 140 m in upwelling regions III and IV are transported to the upper water layer at depths of 20 − 40 m. These two upwelling regions are found to be strongest during summer (especially during July) compared to winter leading to the significant transport of nutrients by 50% from the bottom waters. Therefore, both curl driven upwelling and coastal upwelling can be a major contributor to the nutrients transport to the upper layer as seen in regions III, IV and more pronounced at IV due to high nutrients at the bottom layer. However, less nutrients are upwelled to the upper surface layer at these two regions during winter due to the weak upwelling in which the nutrients could only be transported to depth more than 40 m. As for regions I and II, region II has a uniform nitrate profile around 0.13 μM during both seasons due to the very low concentrations of nutrients at deeper waters and strong curl upwelling causing the well mixed column. As for region I, there is transport of 50% of nitrate from depth 20 m (0.1 μM) to the upper water causing low nitrate content (0.05μM) at depths of 5–10 m. Whereas at both regions I and II, very low phosphate transport (32% rate) is observed from the depths between 20 and 25 m (0.3 μM) to the upper layer depths of 5 −10 m (0.18 μM) during both seasons. However, surface waters at the upwelling regions I and II receive additional silicate, which may be attributed to the presence of the dominant larger species like diatoms in the Arabian Gulf. To further understand the effect of upwelling on the nutrient’s concentrations, the concentrations of nitrate, phosphate and silicate during summer and winter are extracted for shallow waters (depth of 20 m) at upwelling regions I and II and deeper waters (depth of 140 m) at upwelling regions III and IV. Higher concentrations of nitrate and phosphate are observed during summer compared to winter with increase exceeding 90% in the upwelling region III and 100% in the upwelling region IV at depth 140 m. This significant elevation in nutrient concentrations between the summer and winter seasons, suggests that the strong coastal upwelling is able to create a temperature gradient and supply nutrients to the upper layer particularly at region IV. However, the concentrations of nitrate decrease by 43% and phosphate by 5–15% during summer compared to winter at the upwelling regions I, and II at depth 20 m. This is explained by the oligotrophic conditions of the Arabian Gulf due to the lack of nutrients during winter with minimal effect of coastal upwelling. In addition, the associated cooling effect with the coastal upwelling at regions I (*UIsst* 0.38°C) and II (*UIsst* -0.21°C) (Table 3) seems to be insignificant. These results have been confirmed with the correlations between the nutrients and upwelling vertical velocity (*wcoast* and *wcurl*) in addition to SST. Surface waters of the Arabian Gulf have shown a high coefficient of determination (R^2^) between phosphate and coastal upwelling (0.53), as well as between phosphate and SST (R^2^ of 0.36). Similarly, high R^2^ is observed between silicate and coastal upwelling (R^2^ is 0.4). In contrast, all other nutrients have not shown significant statistical correlations with upwelling vertical velocities (Table 4). The R^2^ values for the sea of Oman, however, are found to be very high, particularly in the surface waters. The correlation between coastal upwelling and nitrate is 0.48, phosphate is 0.52 and silicate is 0.36, which is higher than the correlation between these nutrients with curl-driven upwelling (R^2^ < 0.27). In contrast, R^2^ for deep concentrations of nutrients and upwelling at 140 m does not show a strong correlation (Table 5).

**Table 3:** Mean SST upwelling index, Ekman layer depth, vertical velocity of curl-driven upwelling, vertical velocity of coastal upwelling, total vertical velocity, and nutrients concentrations and nutrients concentrations extracted at a depth: ~20m in both I and II and depth: ~140m at III and IV at the upwelling regions during the winter season (Dec, Jan, Feb) for the year 2018. Ekman layer depth and vertical velocity are derived from the monthly climatology of wind datasets which are extracted from the ECMWF (ERA5) datasets for the year 2018. Climatologies have been calculated over the long-term average period of 1981–2010. Whereas nutrient’s data are downloaded from the monthly climatology of the WOA data for the year 2018. The WOA data are climatological data collected from the periods 1900–2017.

Upwelling region	UI_SST_ (°C)	Ekman layer depth (m)	w_curl_ (10^-5^m s^-1^)	w_coast_ (10^-5^m s^-1^)	Total vertical velocity (10^−5^ m s^-1^)	Nitrate (μM)	Phosphate (μM)	Silicate (μM)	N:P ratio
I	1.02	49	0.62	-0.026	0.59	0.26	0.19	0.51	1.4:1
II	0.99	42	0.64	-0.015	0.62	0.28	0.20	0.36	1.4:1
III	-0.16	20	0.044	-0.0009	0.043	1.76	0.46	6.57	3.8:1
IV	0.21	7.5	-0.02	0.0028	-0.017	2.56	0.56	16.7	4.6:1

**Table 4:** Coefficient of determination (R^2^) and its corresponding-significance test p-values, between all pairs of variables: Nutrients (nitrate, phosphate, silicate), vertical velocity of coastal upwelling (w_curl_), vertical velocity of curl-driven upwelling (w_coast_), sea surface temperature (SST) for: 1) the entire region of the Arabian gulf, 2) at depth of 20m of the upwelling region.

**Entire Surface**	**Nitrate**		**Phosphate**		**Silicate**	
	**R^2^**	**P-value**	**R^2^**	**P-value**	**R^2^**	**P-value**
**w**_**curl**_	0.0003	0.00001	0.008	4.7E-112	0.03	3.4E-252
**w**_**coast**_	0.00001	0.9	0.53	<0.05	0.4	<0.05
**SST**	0.002	1E-21	0.36	<0.05	0.4	<0.05
**Depth 20m-upwelling region**	**R** ^**2**^	**P-value**	**R** ^**2**^	**P-value**	**R** ^**2**^	**P-value**
**w**_**curl**_	0.13	6E-129	0.14	8.6E-136	0.014	2.04E-14
**w**_**coast**_	0.06	5.74E-58	0.07	1.42E-68	0.12	6.9E-120
**SST**	0.03	5.75E-31	0.008	2.03E-08	0.03	1.02E-33

**Table 5:** Coefficient of determination (R^2^) and its corresponding-significance test p-values, between all pairs of variables: Nutrients (nitrate, phosphate, silicate), vertical velocity of coastal upwelling (w_curl_), vertical velocity of curl-driven upwelling (w_coast_), sea surface temperature (SST) for: 1) the entire region of the Sea of Oman, 2) at the upwelling region, and 3) at depth of 140m of the upwelling region.

**Entire surface**	**Nitrate**		**Phosphate**		**Silicate**	
	**R^2^**	**P-value**	**R^2^**	**P-value**	**R^2^**	**P-value**
**w**_**curl**_	0.15	<0.05	0.38	<0.05	0.27	<0.05
**w**_**coast**_	0.48	<0.05	0.52	<0.05	0.36	<0.05
**SST**	0.44	<0.05	0.52	<0.05	0.4	<0.05
**Depth 140m- upwelling region**	**R** ^**2**^	**P-value**	**R** ^**2**^	**P-value**	**R** ^**2**^	**P-value**
**w**_**curl**_	0.0004	2.58E-05	0.04	<0.05	0.17	<0.05
**w**_**coast**_	0.17	<0.05	0.15	<0.05	0.15	<0.05
**SST**	0.03	7.2E-290	0.02	1.2E-213	0.03	1.9E-239

**Fig 11:**
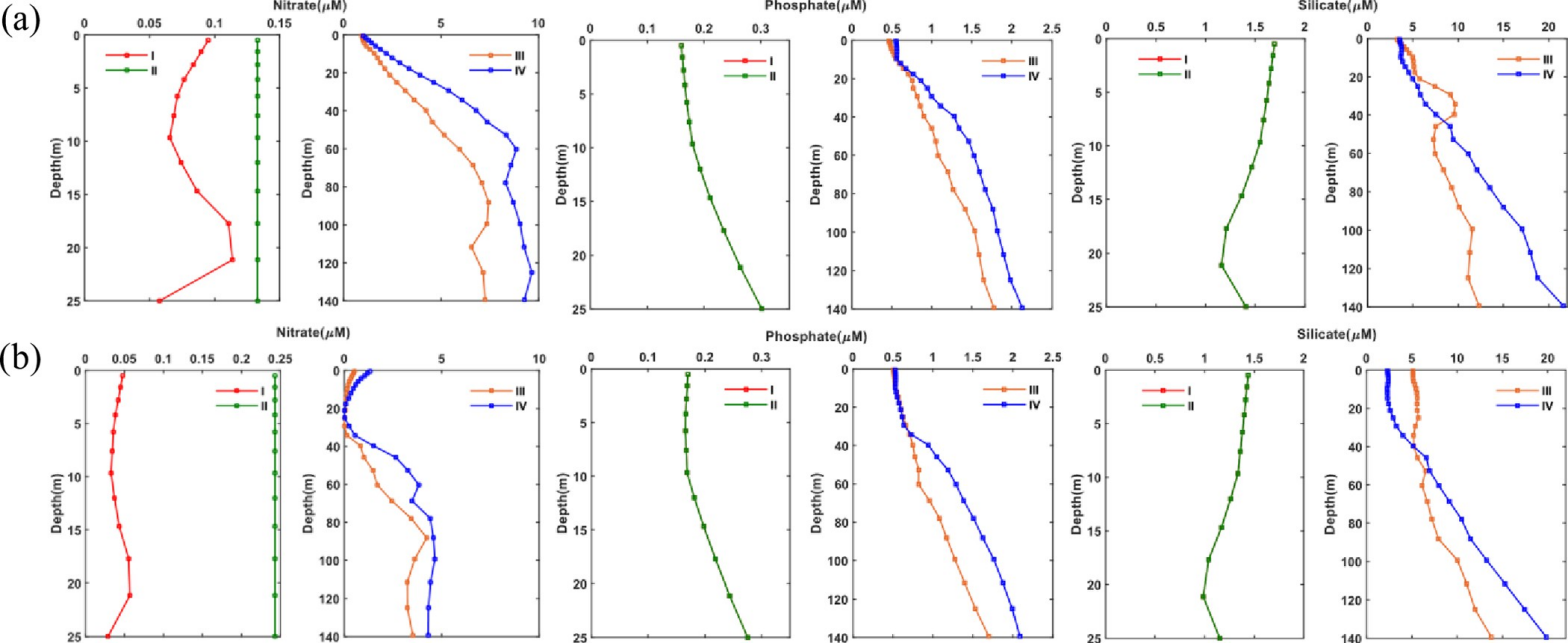
Nutrients (nitrate, phosphate, and silicate) profile at the upwelled waters from the surface up to a depth of 25 m at I and II and depth of 140 at III and IV extracted from the monthly climatology of the World Ocean Atlas data (WOA) for the year 2018. The WOA data are climatological data collected from the periods 1900–2017. Left: Nitrate, middle: Phosphate and right: Silicate. a): Summer (June, July, August); b): Winter (December, January, February).

Although, the concentrations of nutrients are found to be higher at the upwelling regions compared to elsewhere, they still show low N:P ratios compared to the Redfield ratio during summer (≤ 3.8: 1) and winter (≤ 4.6:1) as shown in Table 3. Similarly, silicate concentrations have shown variations of an increase by 6 −12% at regions I, II and IV during summer compared to winter but a slight decrease by 10% at region III. This can be attributed to the significant impact of upwelling conditions as well as the strong cooling effect in regions III and IV. See Fig 11. However, the concentrations of silicate are low in the Arabian Gulf which is shown in Atlantis II and Meteor measurements in which near surface silicate depletion has been observed (≤ 1 μM) [43]. Based on the observed distribution of nutrients at the Arabian Gulf waters, the upwelling effects on Arabian Gulf waters are less pronounced than the upwelling events in Peru [56], Oregon [57], and California [58], which showed high levels of nutrients. As an example, although upwelling currents on the western Iranian coast are stronger than those on the western and southern coasts of the Arabian Gulf due to the higher kinetic energy and current vectors observed in the Iranian coasts [27], the amount of nutrients in the upwelled waters during the summer is still limited. However, there is significant open-sea upwelling and coastal upwelling observed along Oman’s east coast, which causes strong upwelling and consequent vertical transport of nutrients. In addition, upwelling effects cannot be assessed using surface nutrients since phytoplankton rapidly consume nutrients at the water surface. Both curl driven upwelling and coastal upwelling can be a major contributor to the nutrients transport to the upper layer as seen in regions III, IV and more pronounced at IV due to high nutrients at the bottom layer.

## Conclusions

This study presents the spatial dynamics of nutrients and investigates the average surface and water column concentrations. The average surface nutrient concentrations in the Arabian Gulf and Sea of Oman are higher during winter than during summer, except for nitrates. A very low concentration of nitrates is observed during summer and winter in Arabian Gulf waters, with an average value of 0.14–0.16 μM, while high concentrations are observed in the Sea of Oman in winter. Moreover, both the surface and bottom layers of the Sea of Oman exhibit higher levels of nutrients than the Arabian Gulf and the nutrients become more concentrated as depth increases. Nutrient distribution varies according to upwelling regions in the Arabian Gulf and Sea of Oman. There are four strong upwelling zones found in the Arabian Gulf (Iranian coasts; regions I and II) and in the Sea of Oman (southeast coast and northwest coast; regions III and IV). The strongest total vertical velocity region is found in the regions: IV (maximum total vertical velocity of 2.3 x 10^−5^ m s^-1^ in July), then regions I and II (maximum total vertical velocity of 1 x 10^−5^ m s^-1^ in January). Whereas the least intensity is found at III (maximum total vertical velocity ~0.7 x 10^−6^ m s^-1^ in September). Thus, the Sea of Oman has shown an increase in nitrate and phosphate concentrations at a certain depth of the Ekman layer. There is, however, a slight increase in silicate in both regions. The Arabian Gulf also shows slight vertical variations, whereas the sea of Oman shows greater vertical variations of nutrients. This is explained by the stronger upwelling occurring in the Sea of Oman and the availability of nutrients in the deeper waters of the Sea of Oman allowing vertical transport of nutrients. Further studies on vertical distribution of nutrients during upwelling events in the region would be required in the future to support these findings.

## Supporting information

S1 Appendix (DOCX)

## References

[1] Jónasdóttir SH. Fatty acid profiles and production in marine phytoplankton. Mar Drugs. 2019;17: 151. doi: 10.3390/md17030151 30836652 PMC6471065

[2] Caron DA, Alexander H, Allen AE, Archibald JM, Armbrust EV, Bachy C, et al. Probing the evolution, ecology and physiology of marine protists using transcriptomics. Nat Rev Microbiol. 2017;15: 6–20. doi: 10.1038/nrmicro.2016.160 27867198

[3] Dixon JL. Macro and micro nutrient limitation of microbial productivity in oligotrophic subtropical Atlantic waters. Environ Chem. 2008;5: 135–142.

[4] Hawkesford M, Horst W, Kichey T, Lambers H, Schjoerring J, Møller IS, et al. Functions of macronutrients. Marschner’s mineral nutrition of higher plants. Elsevier; 2012. pp. 135–189.

[5] Eyster C. Micronutrient requirements for green plants, especially algae. Algae and man. Springer; 1964. pp. 86–119.

[6] Mooij PR, de Jongh LD, van Loosdrecht MCM, Kleerebezem R. Influence of silicate on enrichment of highly productive microalgae from a mixed culture. J Appl Phycol. 2016;28: 1453–1457. doi: 10.1007/s10811-015-0678-2 27226699 PMC4851980

[7] Okcu GD, Eustance E, Lai YS, Rittmann BE. Evaluation of co-culturing a diatom and a coccolithophore using different silicate concentrations. Sci Total Environ. 2021;769: 145217. doi: 10.1016/j.scitotenv.2021.145217 33493907

[8] Moore CM, Mills MM, Arrigo KR, Berman-Frank I, Bopp L, Boyd PW, et al. Processes and patterns of oceanic nutrient limitation. Nat Geosci. 2013;6: 701–710.

[9] Mock T, Thomas DN. Microalgae in polar regions: linking functional genomics and physiology with environmental conditions. Psychrophiles: from biodiversity to biotechnology. Springer; 2008. pp. 285–312.

[10] Behrenfeld MJ, Falkowski PG. Photosynthetic rates derived from satellite-based chlorophyll concentration. Limnol Oceanogr. 1997. doi: 10.4319/lo.1997.42.1.0001

[11] Ærtebjerg G, Andersen JH, Hansen OS. Nutrients and eutrophication in Danish marine waters. A Chall Sci Manag Natl Environ Res Inst. 2003; 126.

[12] ROPME. State Of The Marine Environment Report 2003. 2003;53: 1–217.

[13] Chadwick OA, Derry LA, Vitousek PM, Huebert BJ, Hedin LO. Changing sources of nutrients during four million years of ecosystem development. Nature. 1999;397: 491–497.

[14] Hendry K, Romero O, Pashley V. Nutrient utilization and diatom productivity changes in the low-latitude south-eastern Atlantic over the past 70 ka: response to Southern Ocean leakage. Clim Past. 2021;17: 603–614.

[15] Devlin MJ, Massoud MS, Hamid SA, Al-zaidan A, Al-sarawi H, Al-enezi M, et al. Changes in the water quality conditions of Kuwait ‘ s marine waters: Long term impacts of nutrient enrichment. MPB. 2015;100: 607–620. doi: 10.1016/j.marpolbul.2015.10.022 26490407

[16] Al-ansari EMAS, Rowe G, Abdel-moati MAR, Yigiterhan O, Al-maslamani I, Al-yafei MA, et al. Estuarine, Coastal and Shelf Science Hypoxia in the central Arabian Gulf Exclusive Economic Zone (EEZ) of Qatar during summer season. Estuar Coast Shelf Sci. 2015;159: 60–68. doi: 10.1016/j.ecss.2015.03.022

[17] Ghaemi M, Abtahi B, Gholamipour S. Spatial distribution of nutrients and chlorophyll a across the Persian Gulf and the Gulf of Oman. Ocean Coast Manag. 2021;201: 105476. doi: 10.1016/j.ocecoaman.2020.105476

[18] Wolff GA. Productivity of the oceans: Present and past, edited by Berger W. H., Smetacek V. S. and Wefer G., Report on the Dahlem Workshop on Productivity of the Oceans, Berlin 1988. Wiley. 1991; 100.

[19] Al-Yamani F, Naqvi SWA. Chemical oceanography of the Arabian Gulf. Deep Sea Res Part II Top Stud Oceanogr. 2019;161: 72–80.

[20] Al-Hemoud A, Al-Sudairawi M, Neelamanai S, Naseeb A, Behbehani W. Socioeconomic effect of dust storms in Kuwait. Arab J Geosci. 2017;10. doi: 10.1007/s12517-016-2816-9

[21] Kämpf J, Sadrinasab M. The circulation of the Persian Gulf: a numerical study. Ocean Sci Discuss. 2005;2: 129–164. doi: 10.5194/osd-2-129-2005

[22] Al-Shehhi MR, Nelson D, Farzanah R, Alshihi R, Salehi-Ashtiani K. Characterizing algal blooms in a shallow & a deep channel. Ocean Coast Manag. 2021;213: 105840.

[23] Benazzouz A, Mordane S, Orbi A, Chagdali M, Hilmi K, Atillah A, et al. An improved coastal upwelling index from sea surface temperature using satellite-based approach—The case of the Canary Current upwelling system. Cont Shelf Res. 2014;81: 38–54. doi: 10.1016/j.csr.2014.03.012

[24] Andrews J, Gentien P. Upwelling as a Source of Nutrients for the Great Barrier Reef Ecosystems: A Solution to Darwin’s Question? Mar Ecol Prog Ser. 1982;8: 257–269. doi: 10.3354/meps008257

[25] Watanabe TK, Watanabe T, Yamazaki A, Pfei M, Claereboudt MR. Past summer upwelling events in the Gulf of Oman derived from a coral geochemical record. 2017; 1–7. doi: 10.1038/s41598-017-04865-5 28676643 PMC5496871

[26] Narvekar J, Chowdhury RR, Gaonkar D, Kumar PKD. Observational evidence of stratification control of upwelling and pelagic fishery in the eastern Arabian Sea. Sci Rep. 2021; 1–13. doi: 10.1038/s41598-021-86594-4 33790329 PMC8012587

[27] Mehrfar H, Azad MT, Lari K, Bidokhti AAAA. A numerical simulation case study of the coastal currents and upwelling in the western Persian Gulf. J Ocean Eng Sci. 2020;5: 323–332. doi: 10.1016/j.joes.2019.12.005

[28] Farmanara M, Malakooti H, Hassanzadeh S. Estuarine, Coastal and Shelf Science Large eddy simulation of the Ekman transport in a strati fi ed coastal sea: A case study of the Persian Gulf. Estuar Coast Shelf Sci. 2018;212: 372–386. doi: 10.1016/j.ecss.2018.08.003

[29] Rykaczewski RR, Checkley DM. Influence of ocean winds on the pelagic ecosystem in upwelling regions. Proc Natl Acad Sci U S A. 2008;105: 1965–1970. doi: 10.1073/pnas.0711777105 18250305 PMC2538866

[30] Garcia H.E., Boyer TP, Baranova OK, Locarnini RA, Mishonov AV, Grodsky A, et al. WORLD OCEAN ATLAS 2018 Product Documentation. 2018;1: 1–20. doi: 10.13140/RG.2.2.34758.01602

[31] Aumont O, Ethé C, Tagliabue A, Bopp L, Gehlen M. PISCES-v2: an ocean biogeochemical model for carbon and ecosystem studies. Geosci Model Dev Discuss. 2015;8: 1375–1509. doi: 10.5194/gmdd-8-1375-2015

[32] Dutkiewicz S, Hickman AE, Jahn O, Gregg WW, Mouw CB, Follows MJ. Capturing optically important constituents and properties in a marine biogeochemical and ecosystem model. Biogeosciences. 2015;12: 4447–4481. doi: 10.5194/bg-12-4447-2015

[33] Guieu C, Al Azhar M, Aumont O, Mahowald N, Levy M, Ethé C, et al. Major Impact of Dust Deposition on the Productivity of the Arabian Sea. Geophys Res Lett. 2019;46: 6736–6744. doi: 10.1029/2019GL082770

[34] Jung HC, Moon BK, Wie J, Park HS, Lee J, Byun YH. A single-column ocean biogeochemistry model (GOTM-TOPAZ) version 1.0. Geosci Model Dev. 2019;12: 699–722. doi: 10.5194/gmd-12-699-2019

[35] Krumhardt KM, Lovenduski NS, Long MC, Levy M, Lindsay K, Moore JK, et al. Coccolithophore Growth and Calcification in an Acidified Ocean: Insights From Community Earth System Model Simulations. J Adv Model Earth Syst. 2019;11: 1418–1437. doi: 10.1029/2018MS001483

[36] Lachkar Z, Lévy M, Smith KS. Strong Intensification of the Arabian Sea Oxygen Minimum Zone in Response to Arabian Gulf Warming. Geophys Res Lett. 2019;46: 5420–5429. doi: 10.1029/2018GL081631

[37] Le Quéré C, Buitenhuis ET, Moriarty R, Alvain S, Aumont O, Bopp L, et al. Role of zooplankton dynamics for Southern Ocean phytoplankton biomass and global biogeochemical cycles. Biogeosciences. 2016;13: 4111–4133. doi: 10.5194/bg-13-4111-2016

[38] Sankar S, Polimene L, Marin L, Menon NN, Samuelsen A, Pastres R, et al. Sensitivity of the simulated Oxygen Minimum Zone to biogeochemical processes at an oligotrophic site in the Arabian Sea. Ecol Modell. 2018;372: 12–23. doi: 10.1016/j.ecolmodel.2018.01.016

[39] Sharada MK, Kalyani Devasena C, Swathi PS. Iron limitation study in the North Indian Ocean using model simulations. J Earth Syst Sci. 2020;129. doi: 10.1007/s12040-020-1361-9

[40] Tjiputra JF, Roelandt C, Bentsen M, Lawrence DM, Lorentzen T, Schwinger J, et al. Evaluation of the carbon cycle components in the Norwegian Earth System Model (NorESM). Geosci Model Dev. 2013;6: 301–325. doi: 10.5194/gmd-6-301-2013

[41] Stewart RH. Coastal Procesess and Tides. Introd to Phys Oceanogr. 2008; 353.

[42] KESSLER WS. Mean Three-Dimensional Circulation in the Northeast Tropical Pacific *. 2002; 2457–2471.

[43] Brewer PG, Fleer AP, Kadar S, Shafer DK, Smith CL. Chemical Oceanographic Data from the Persian Gulf and Gulf of Oman. 1978.

[44] Emara H. Nutrient Salts, Inorganic and Organic Carbon Contents in the Waters of the Persian Gulf and the Gulf of Oman. J Persian Gulf. 2010;1: 33–44.

[45] Garcia CA, Baer SE, Garcia NS, Rauschenberg S, Twining BS, Lomas MW, et al. Nutrient supply controls particulate elemental concentrations and ratios in the low latitude eastern Indian Ocean. Nat Commun. 2018;9: 1–10. 30451846 10.1038/s41467-018-06892-w PMC6242840

[46] Shah P, Sajeev R, Thara KJ, George G, Shafeeque M, Akash S, et al. A holistic approach to upwelling and downwelling along the south-west coast of India. Mar Geod. 2019;42: 64–84.

[47] Naqvi SWA, Sarma VVSS, Jayakumar DA. Carbon cycling in the northern Arabian Sea during the northeast monsoon: Significance of salps. Mar Ecol Prog Ser. 2002;226: 35–44. doi: 10.3354/meps226035

[48] Hashimoto S, Tsujimoto R, Maeda M, Ishimaru T, Yoshida J, Takasu Y, et al. Distribution of nutrients, nitrous oxide, and chlorophyll a of RSA: Extremly high ratios of nitrite to nitrate in whole water column. Offshore Environment of the ROPME Sea Area after the War-Related Oil Spill—Results of the 1993–94 Umitaka-Maru Cruises. 1998. pp. 99–124.

[49] Shriadah MA. Nutrient Salts in the United Arab Emirates Waters (the Arabian Gulf and the Gulf of Oman). 2006;15: 1–9.

[50] Chester R. Marine geochemistry. Choice Reviews Online. 1991. doi: 10.5860/choice.28-2743

[51] Al-Said T, Madhusoodhanan R, Pokavanich T, Al-Yamani F, Kedila R, Al-Ghunaim A, et al. Environmental characterization of a semiarid hyper saline system based on dissolved trace metal-macronutrient synergy: A multivariate spatio-temporal approach. Mar Pollut Bull. 2018;129: 846–858. doi: 10.1016/j.marpolbul.2017.10.009 29033172

[52] Chaichitehrani N, Fluid MA-AJ of, 2018 U. Overview of wind climatology for the Gulf of Oman and the northern Arabian Sea. ResearchgateNet. 2018;8: 1–9. doi: 10.5923/j.ajfd.20180801.01

[53] Williams RG, Follows MJ. The Ekman transfer of nutrients and maintenance of new production over the North Atlantic. Deep Res Part I Oceanogr Res Pap. 1998;45: 461–489. doi: 10.1016/S0967-0637(97)00094-0

[54] Gupta R Sen Naqvi SWA. Chemical oceanography of the Indian Ocean, north of the equator. Deep Sea Res Part A, Oceanogr Res Pap. 1984;31: 671–706. doi: 10.1016/0198-0149(84)90035-9

[55] Ghazi E, Bidokhti AA, Ezam M, Azad MT, Hassanzadeh S. Physical Properties of Persian Gulf Outflow Thermohaline Intrusion in the Oman Sea. Open J Mar Sci. 2017;07: 169–190. doi: 10.4236/ojms.2017.71013

[56] Silva N, Rojas N, Fedele A. Water masses in the Humboldt Current System: Properties, distribution, and the nitrate deficit as a chemical water mass tracer for Equatorial Subsurface Water off Chile. Deep Sea Res Part II Top Stud Oceanogr. 2009;56: 1004–1020. doi: 10.1016/j.dsr2.2008.12.013

[57] Van Geen A, Takesue RK, Goddard J, Takahashi T, Barth JA, Smith RL. Carbon and nutrient dynamics during coastal upwelling off Cape Blanco, Oregon. Deep Res Part II Top Stud Oceanogr. 2000;47: 975–1002. doi: 10.1016/S0967-0645(99)00133-2

[58] Timothy Pennington J, Chavez FP. Seasonal fluctuations of temperature, salinity, nitrate, chlorophyll and primary production at station H3/M1 over 1989–1996 in Monterey Bay, California. Deep Res Part II Top Stud Oceanogr. 2000;47: 947–973. doi: 10.1016/S0967-0645(99)00132-0

