# Supplementary material for: Upwelling and nutrient dynamics in the Arabian Gulf and sea of Oman
Source: PLoS One. 2022 Oct 21;17(10):e0276260. doi: 10.1371/journal.pone.0276260 (PMC9586346; doi:10.1371/journal.pone.0276260)
Supplement: S1 Appendix — (DOCX) [file pone.0276260.s001.docx]

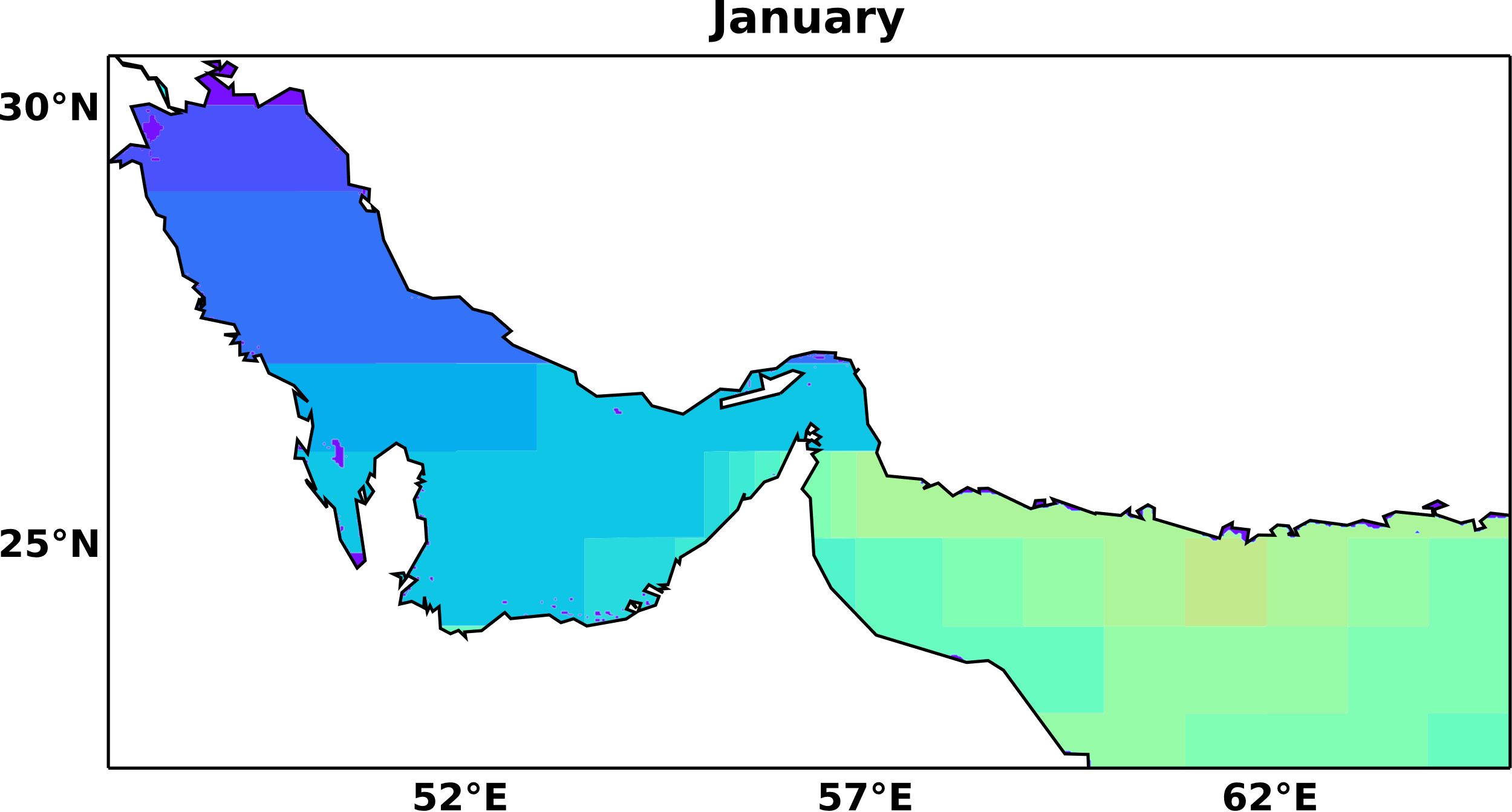

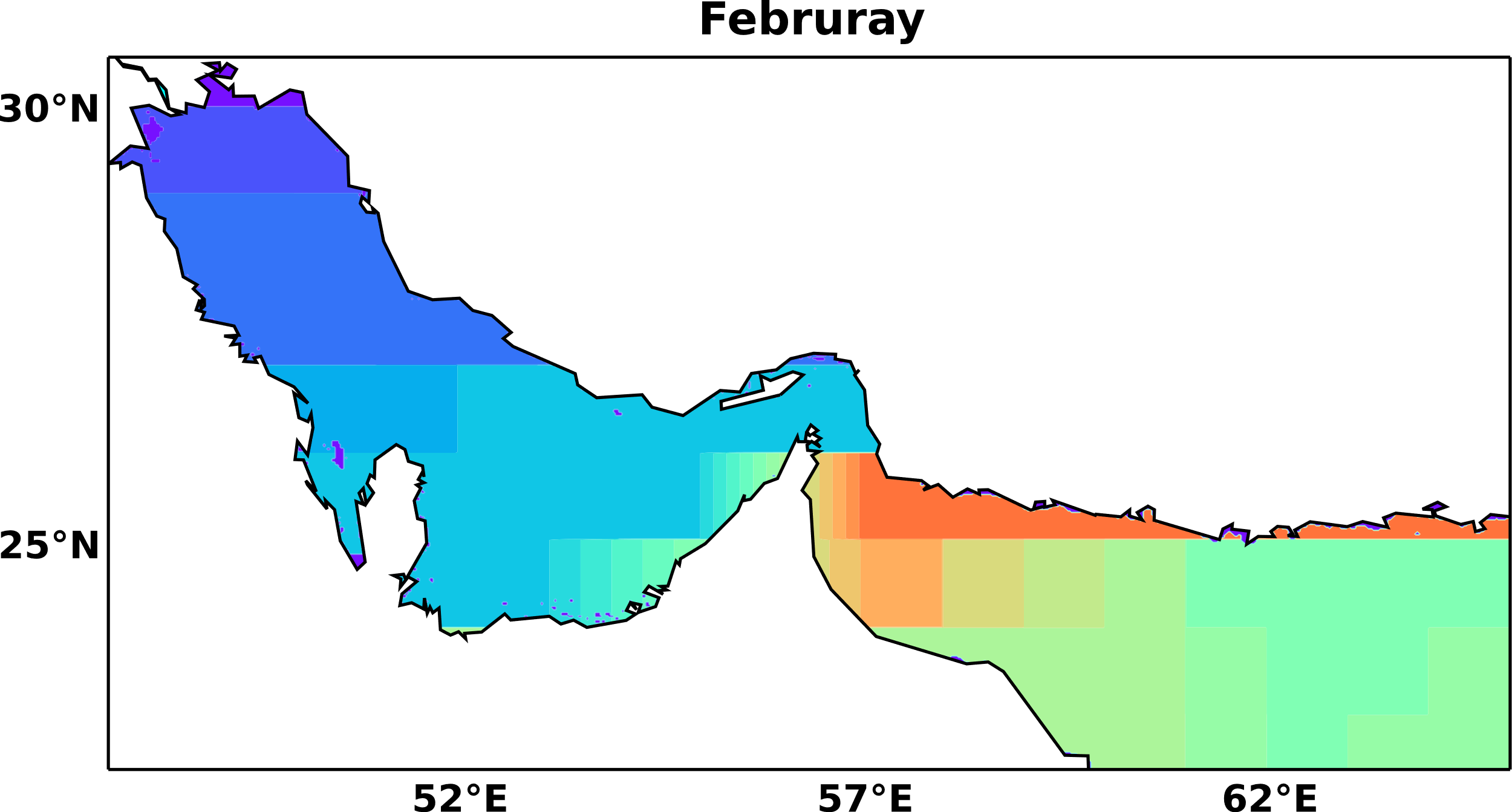

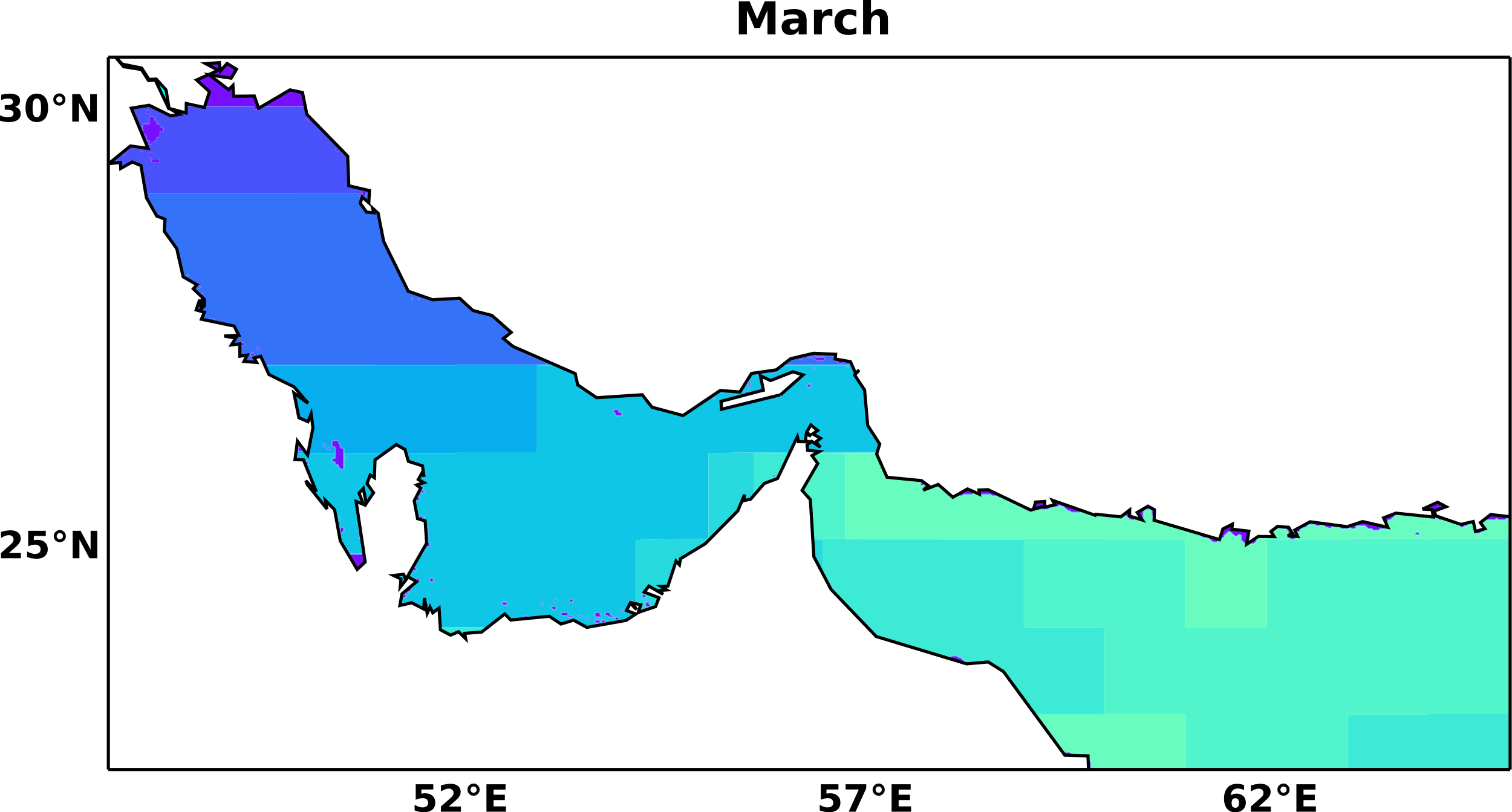

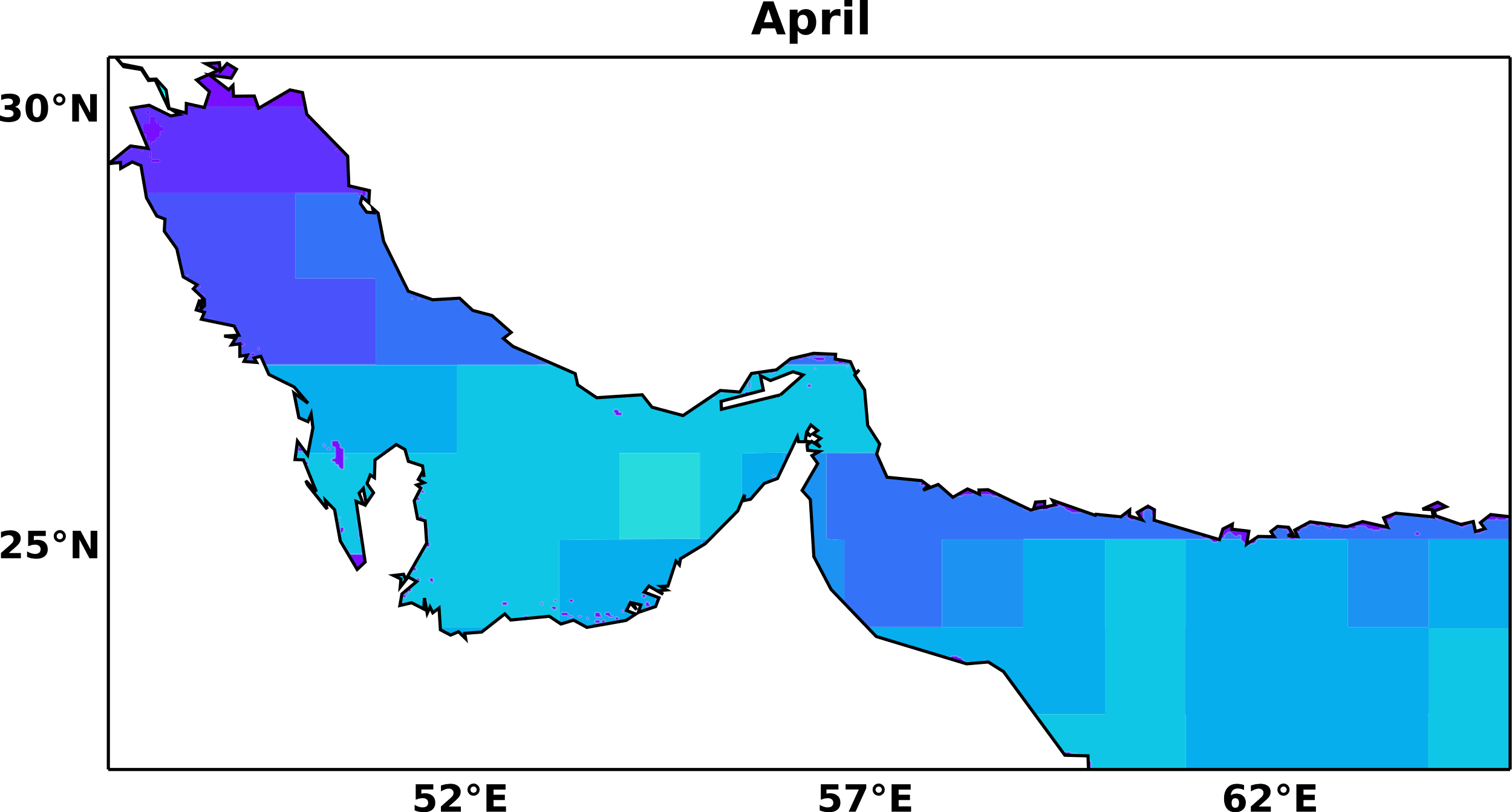

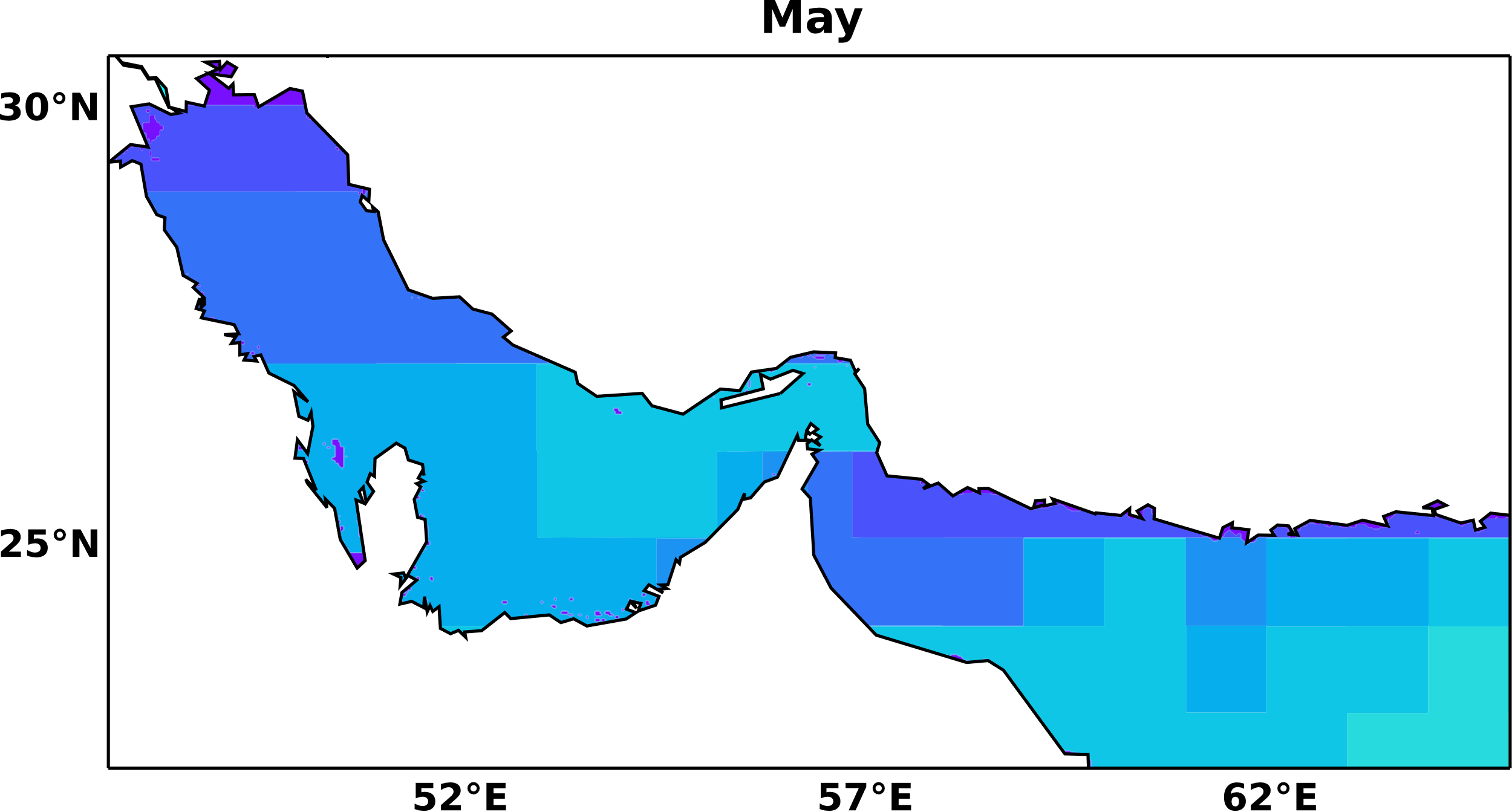

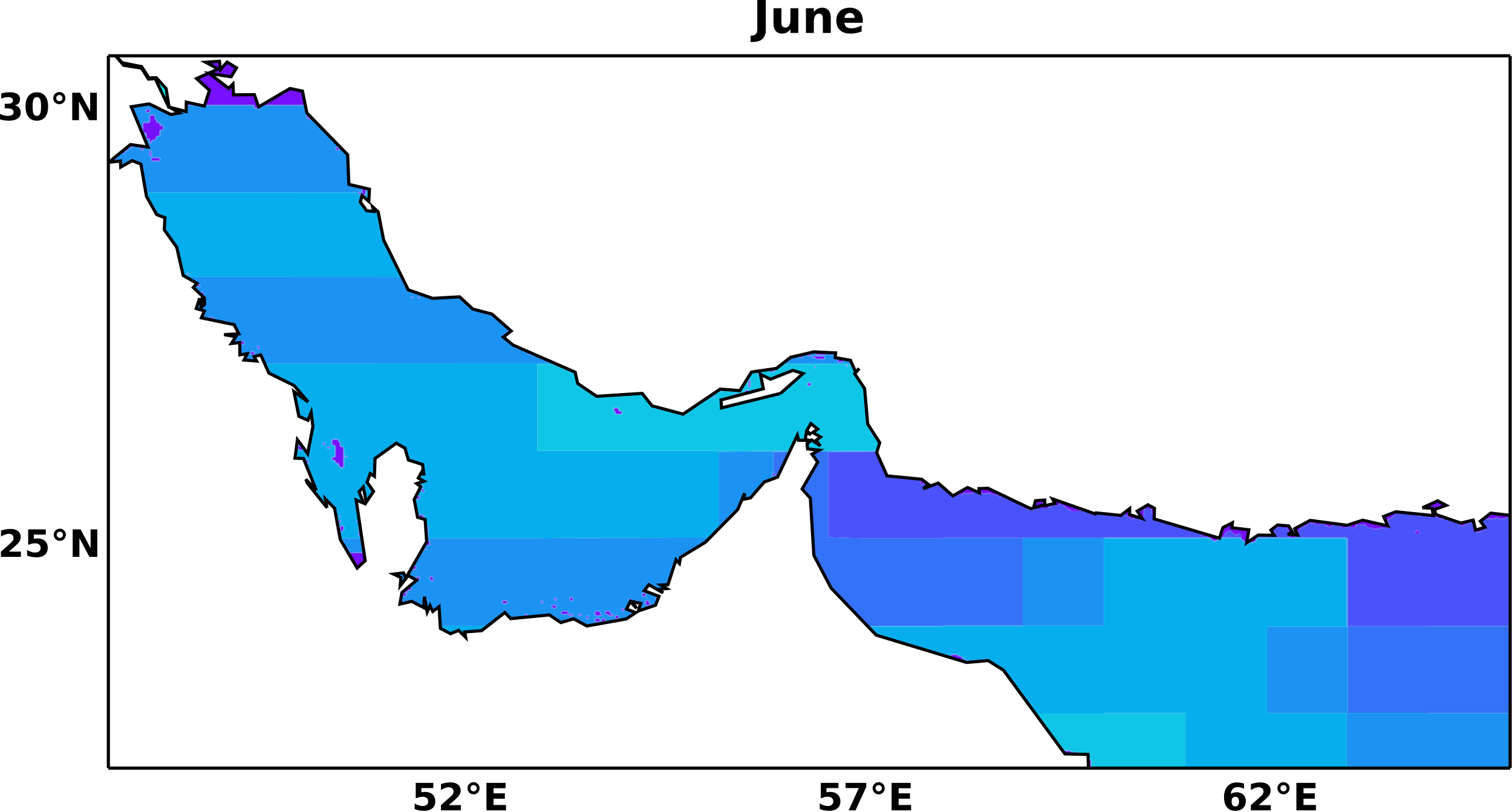

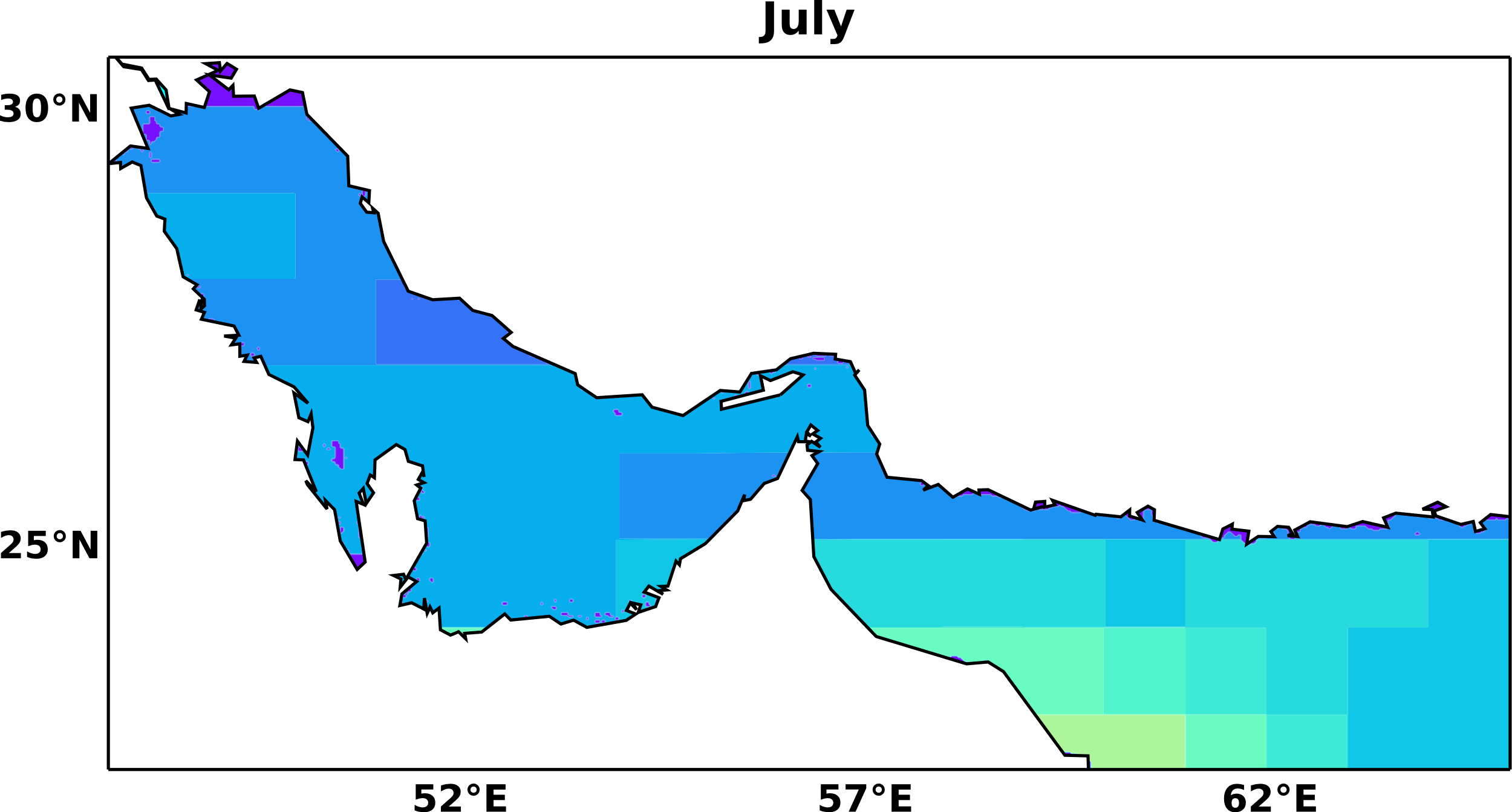

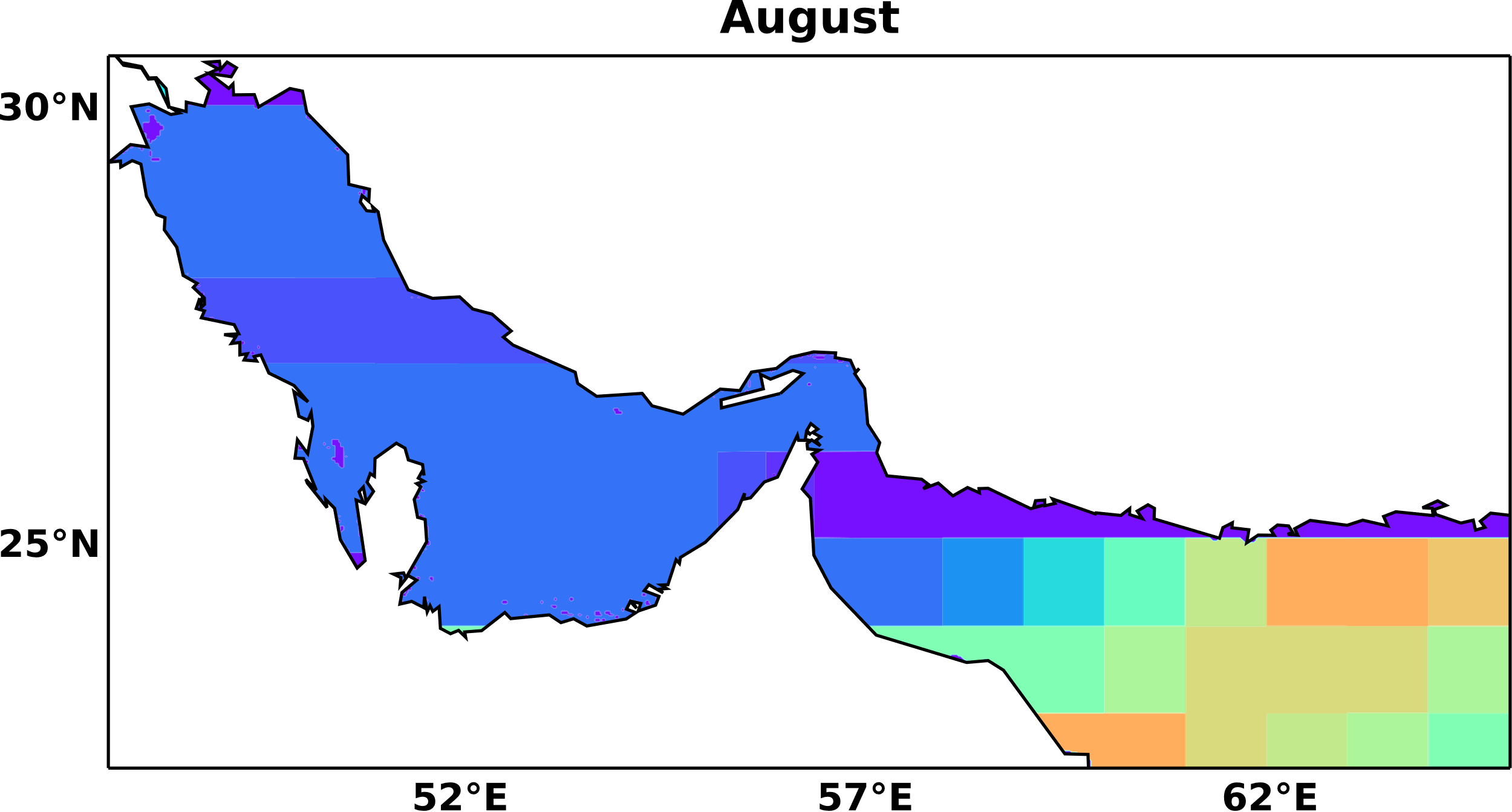

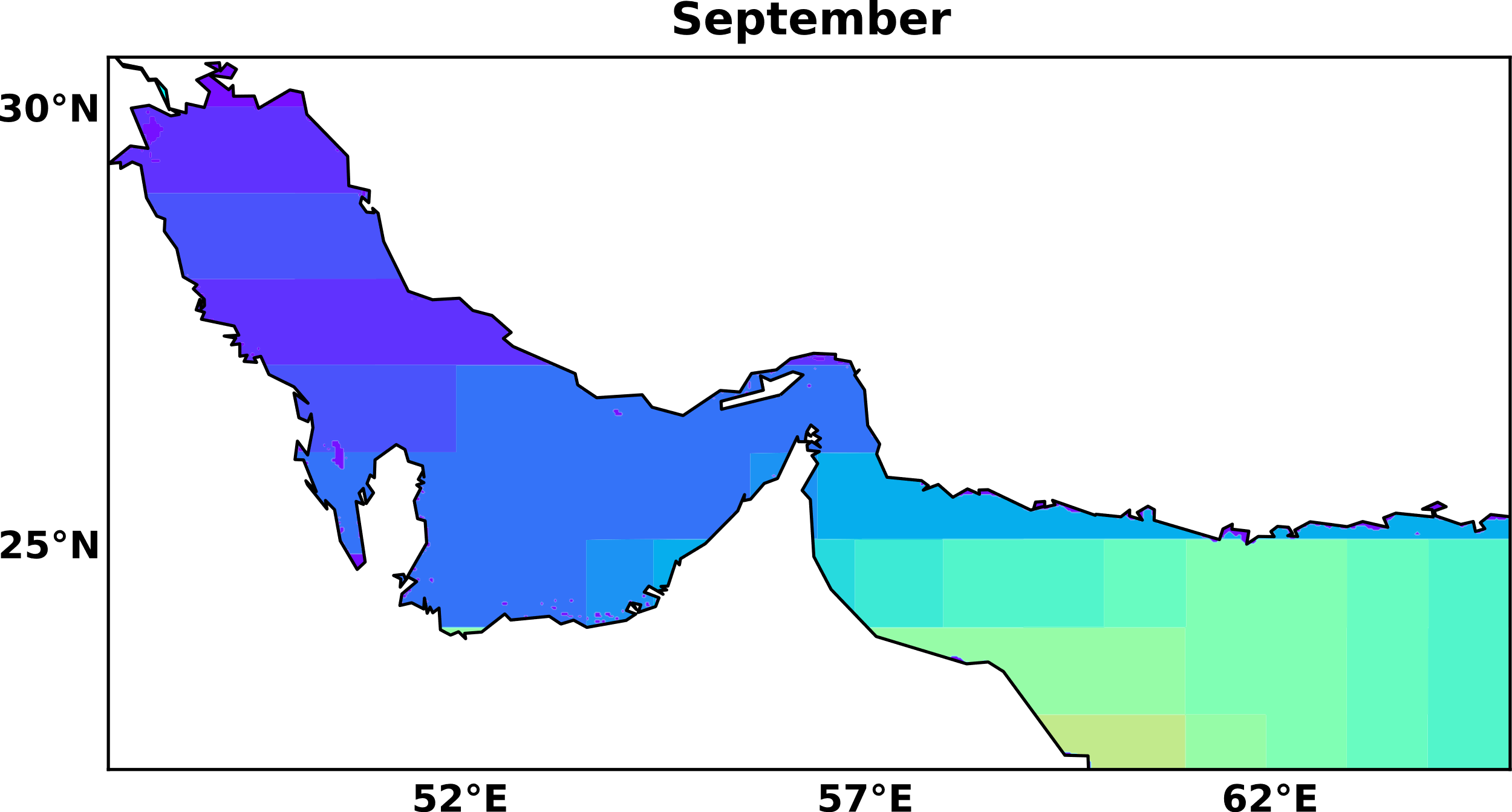

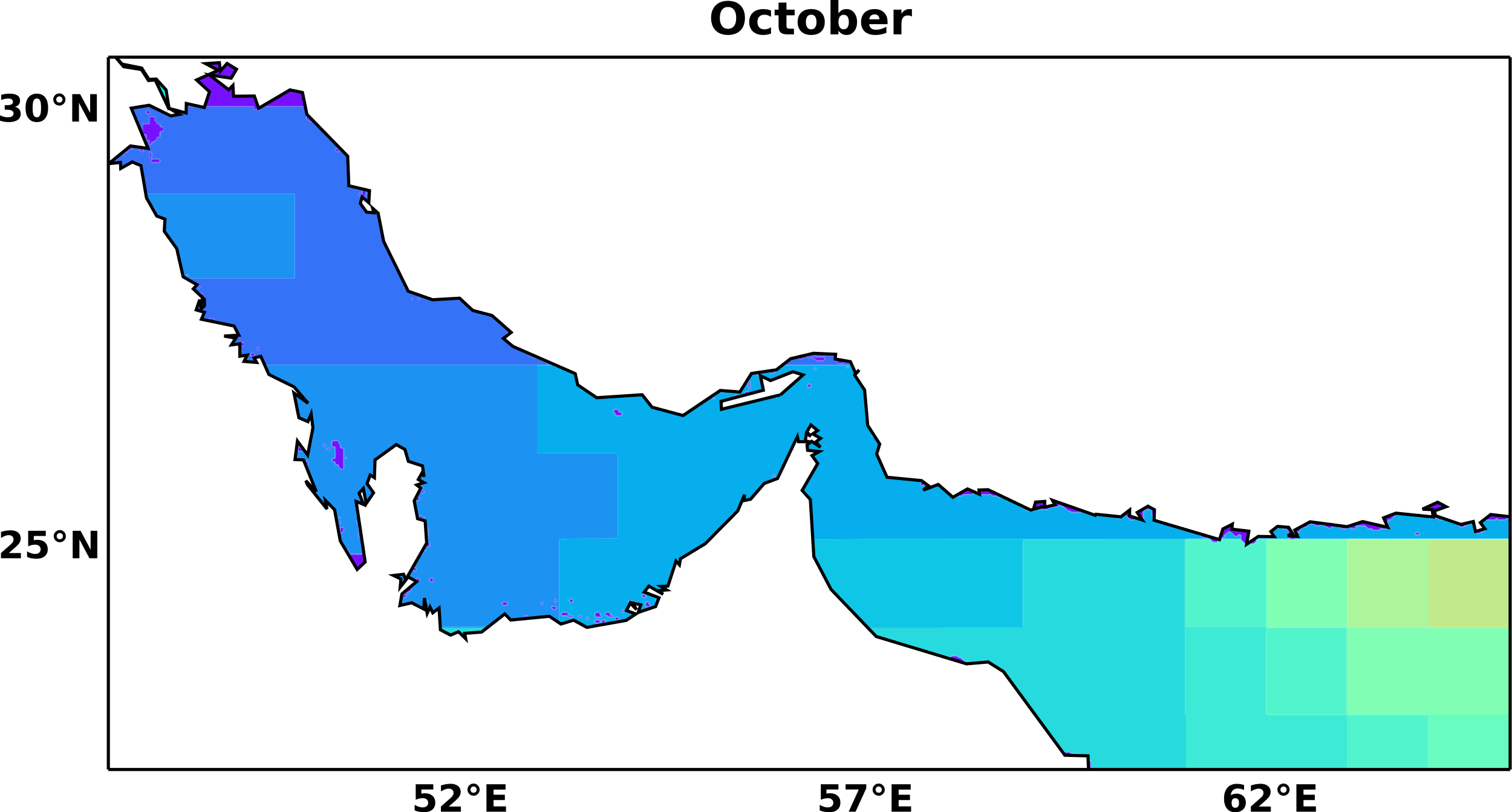

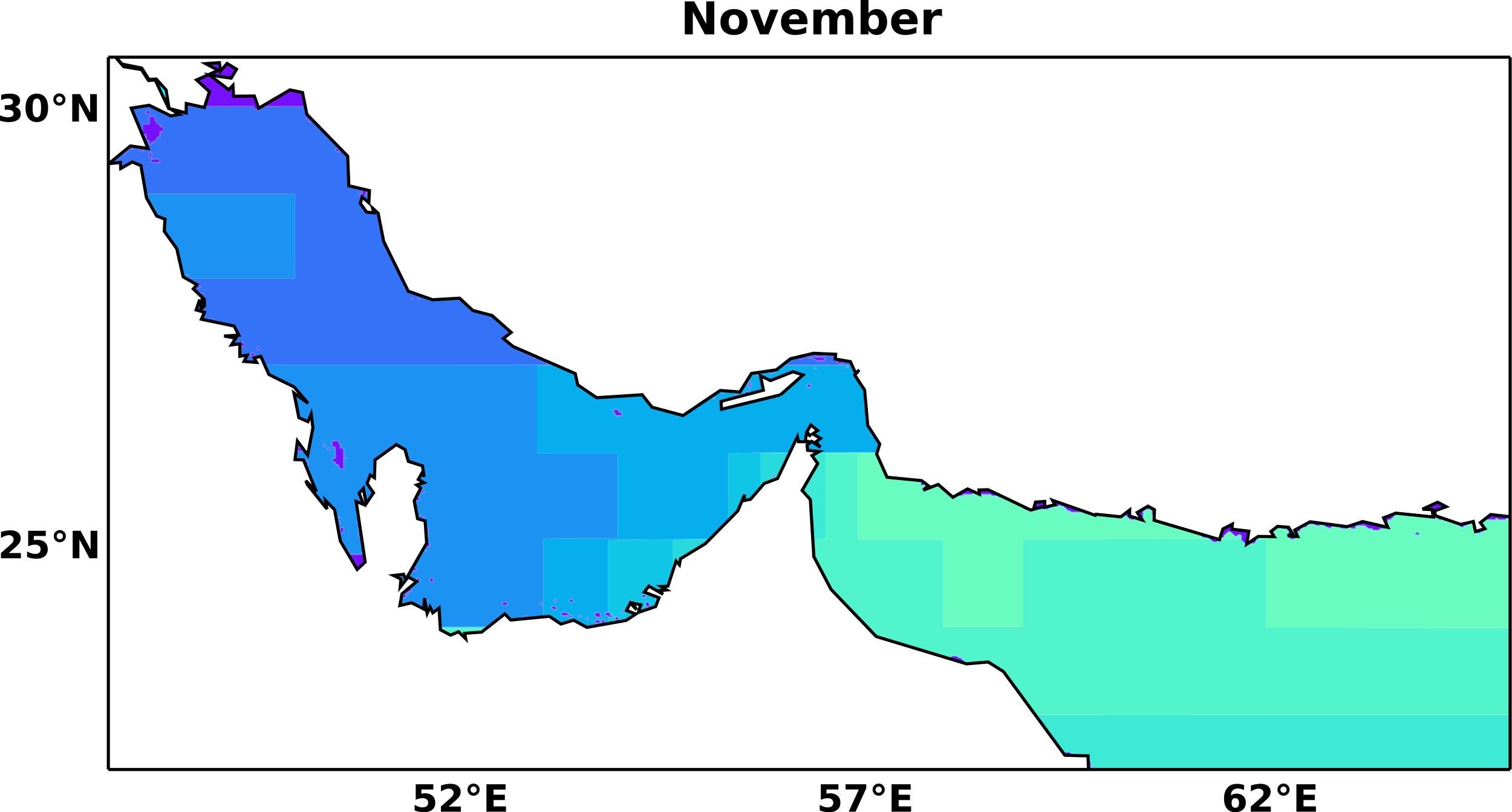

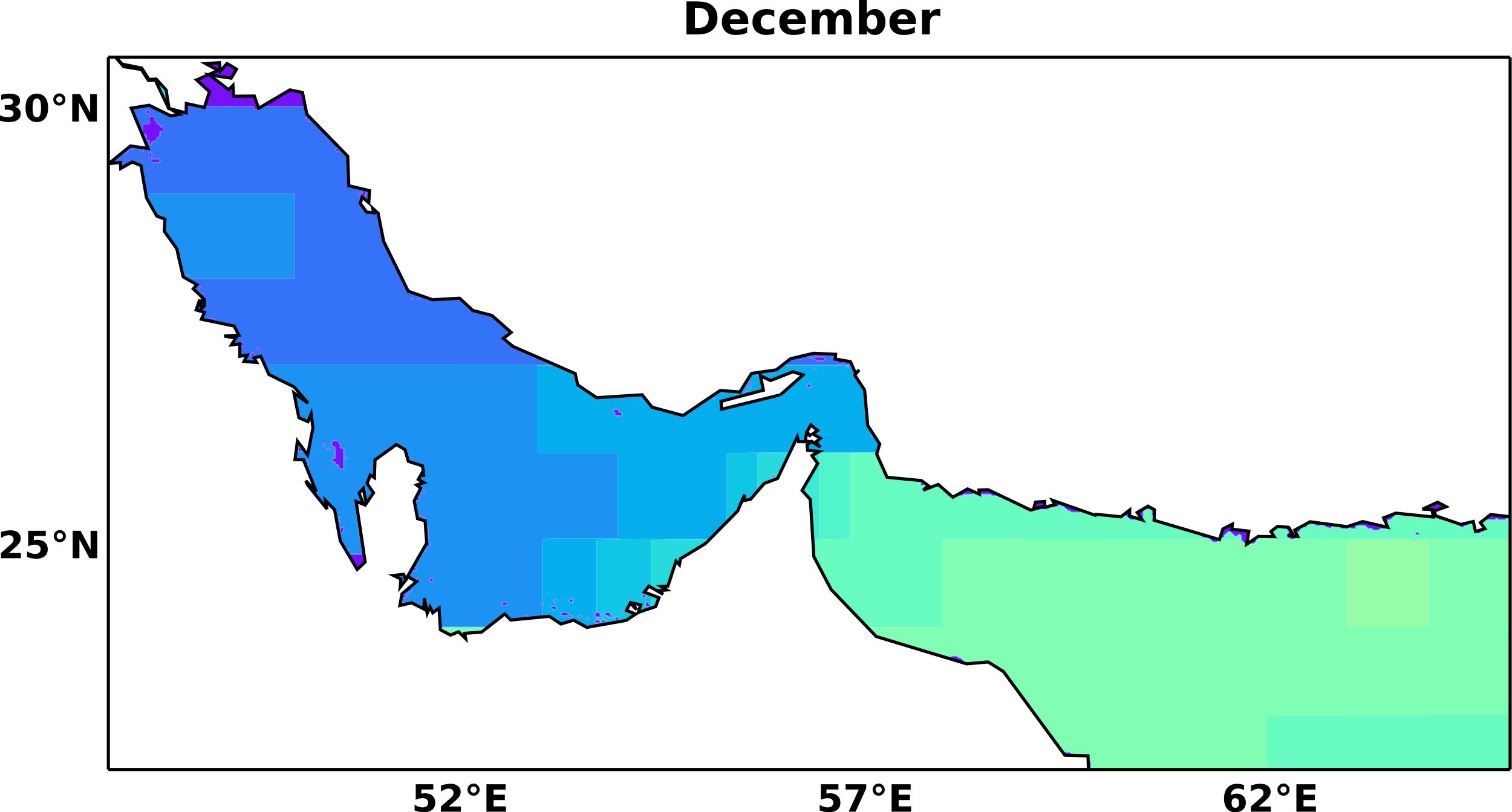


**Phosphate (µM)**


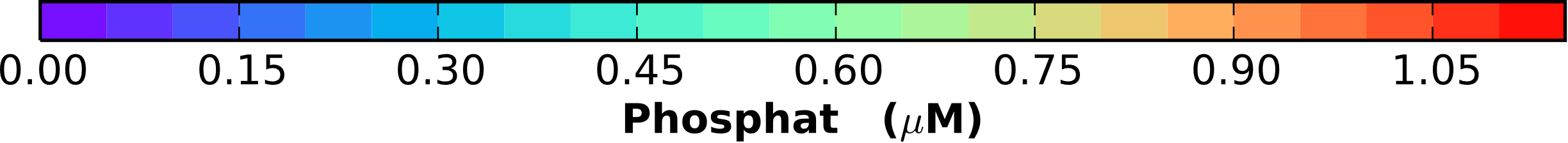


**Fig A.1.** Monthly surface phosphate climatology for the Arabian Gulf and Sea of Oman extracted from the World Ocean Atlas data (WOA) for the year 2018.


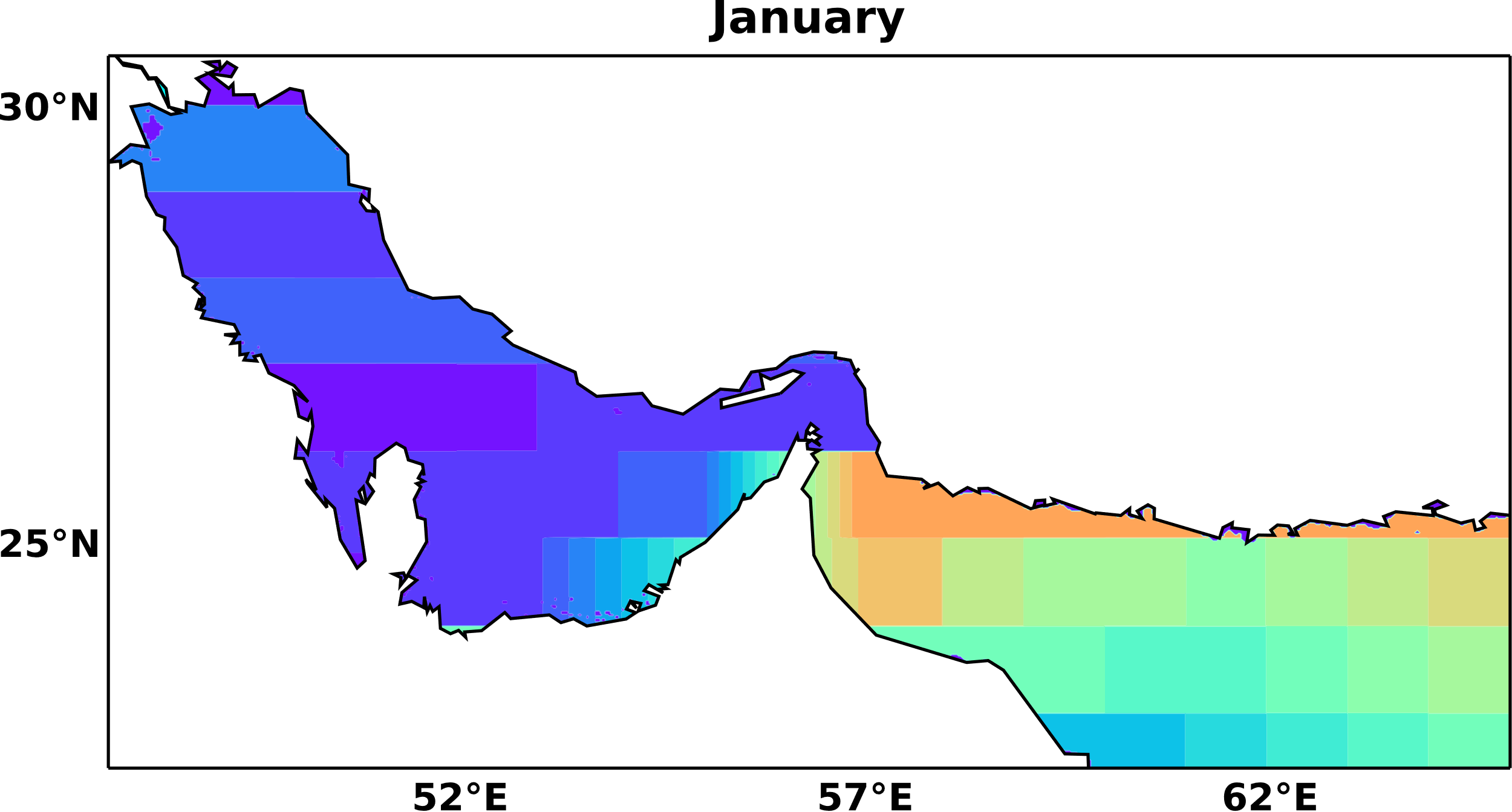

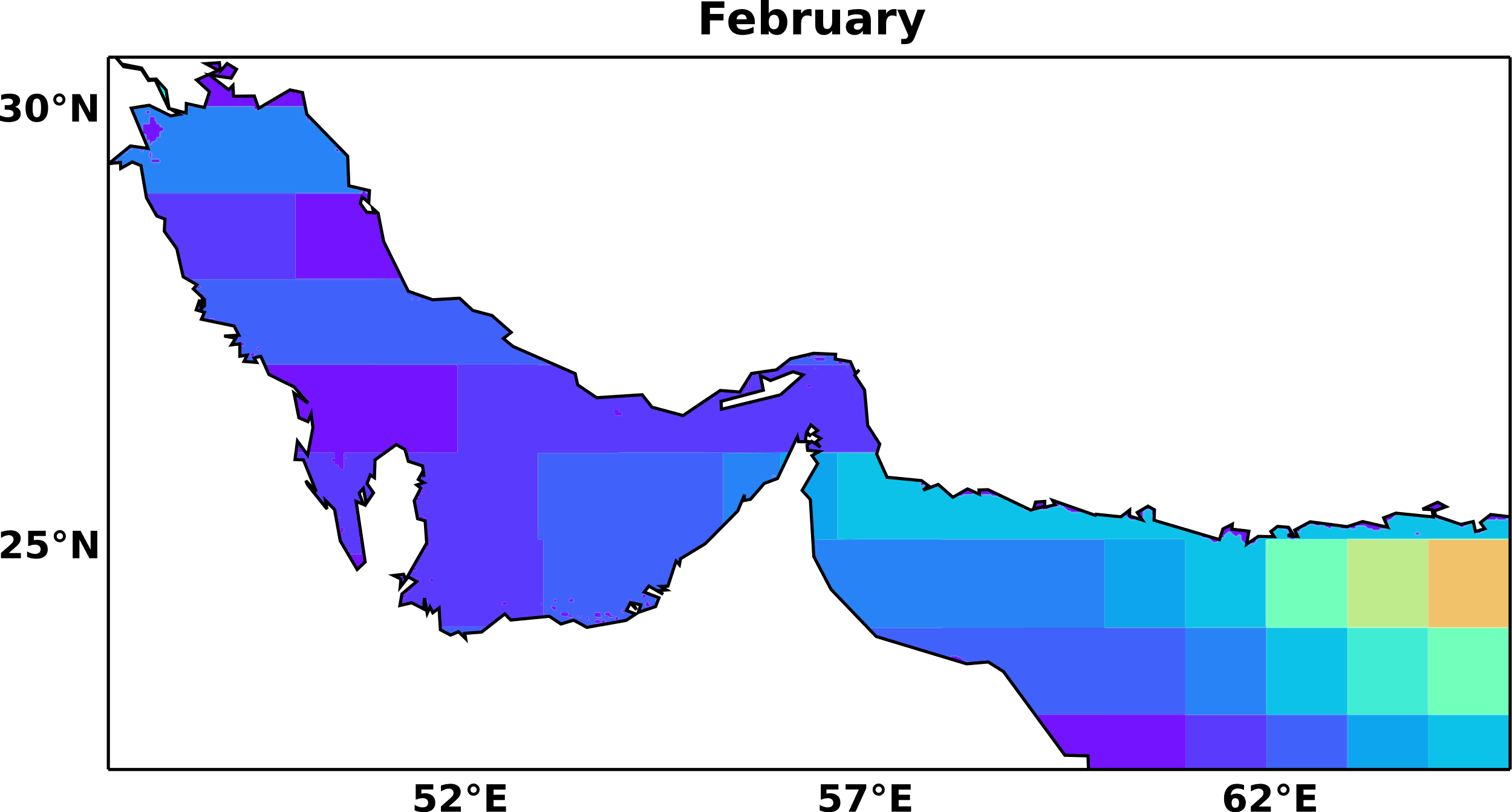

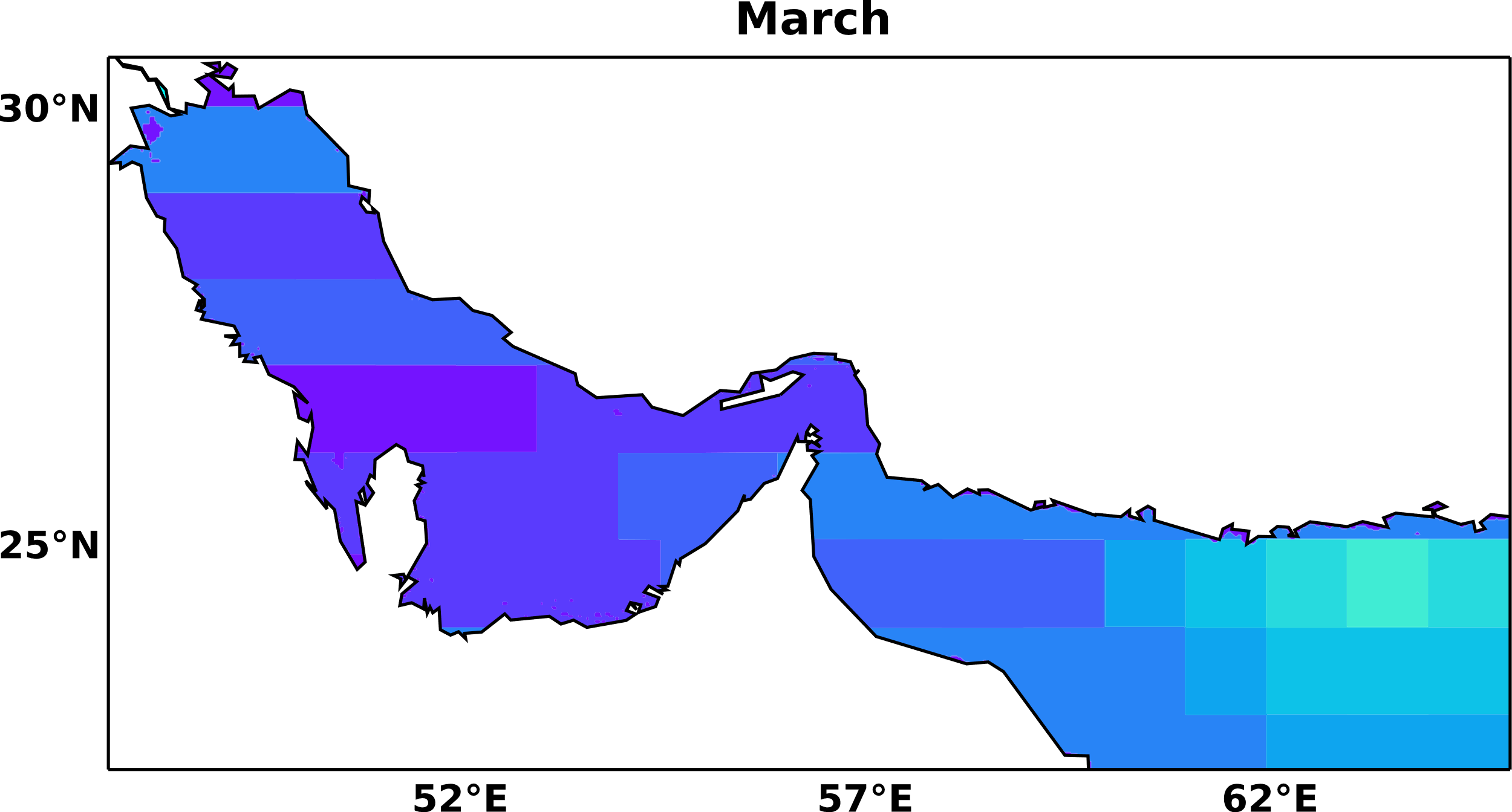

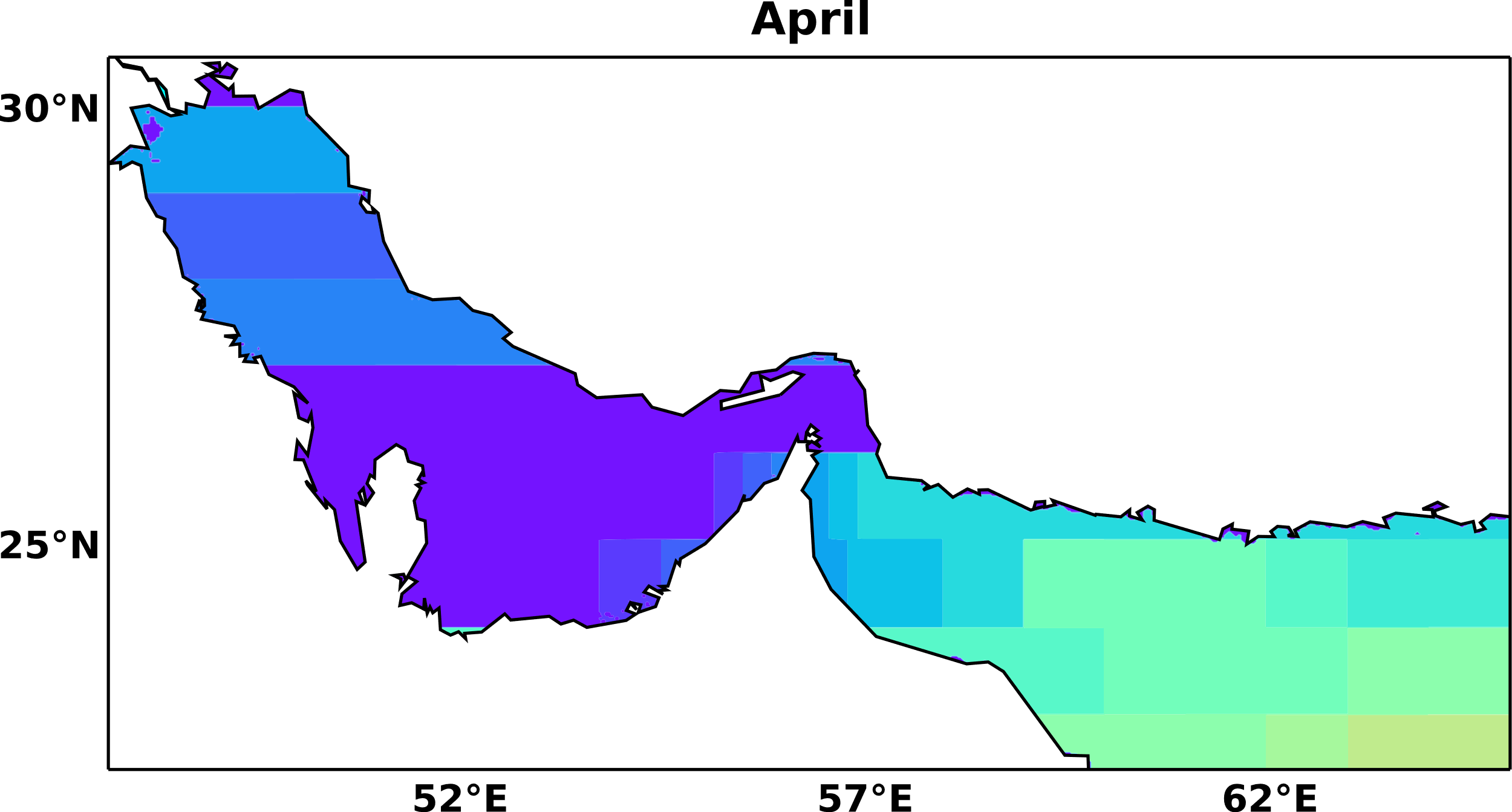

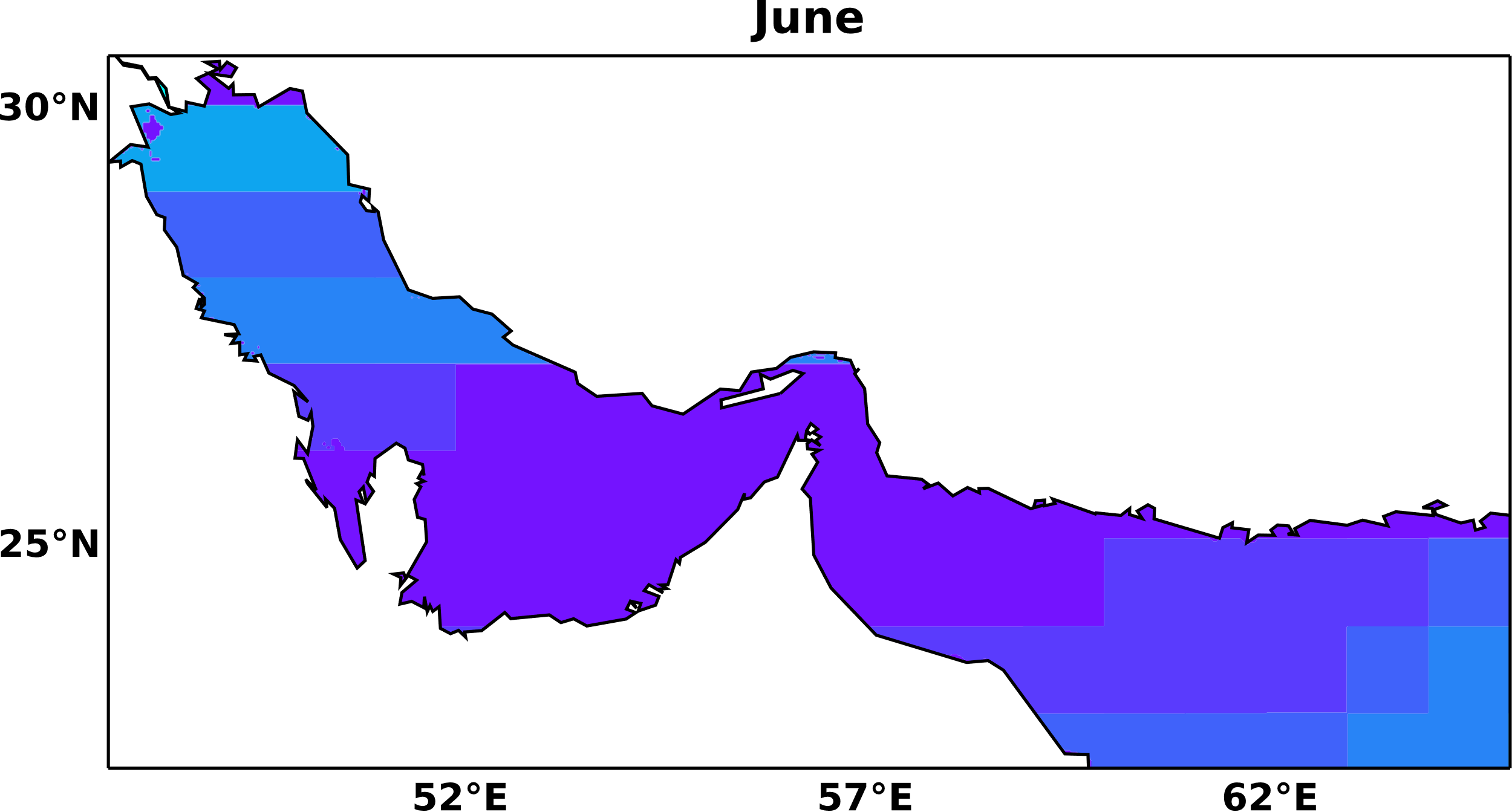

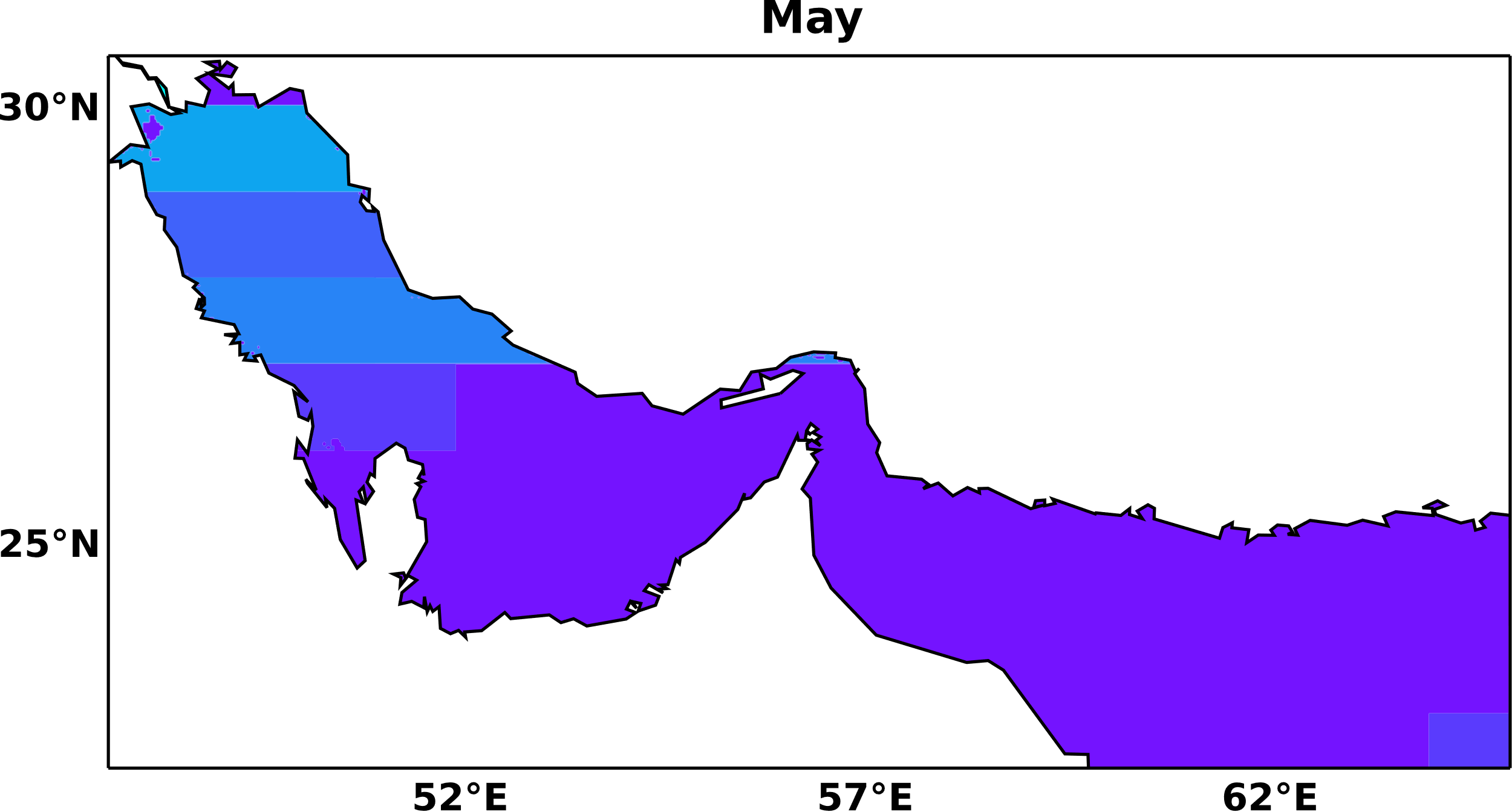

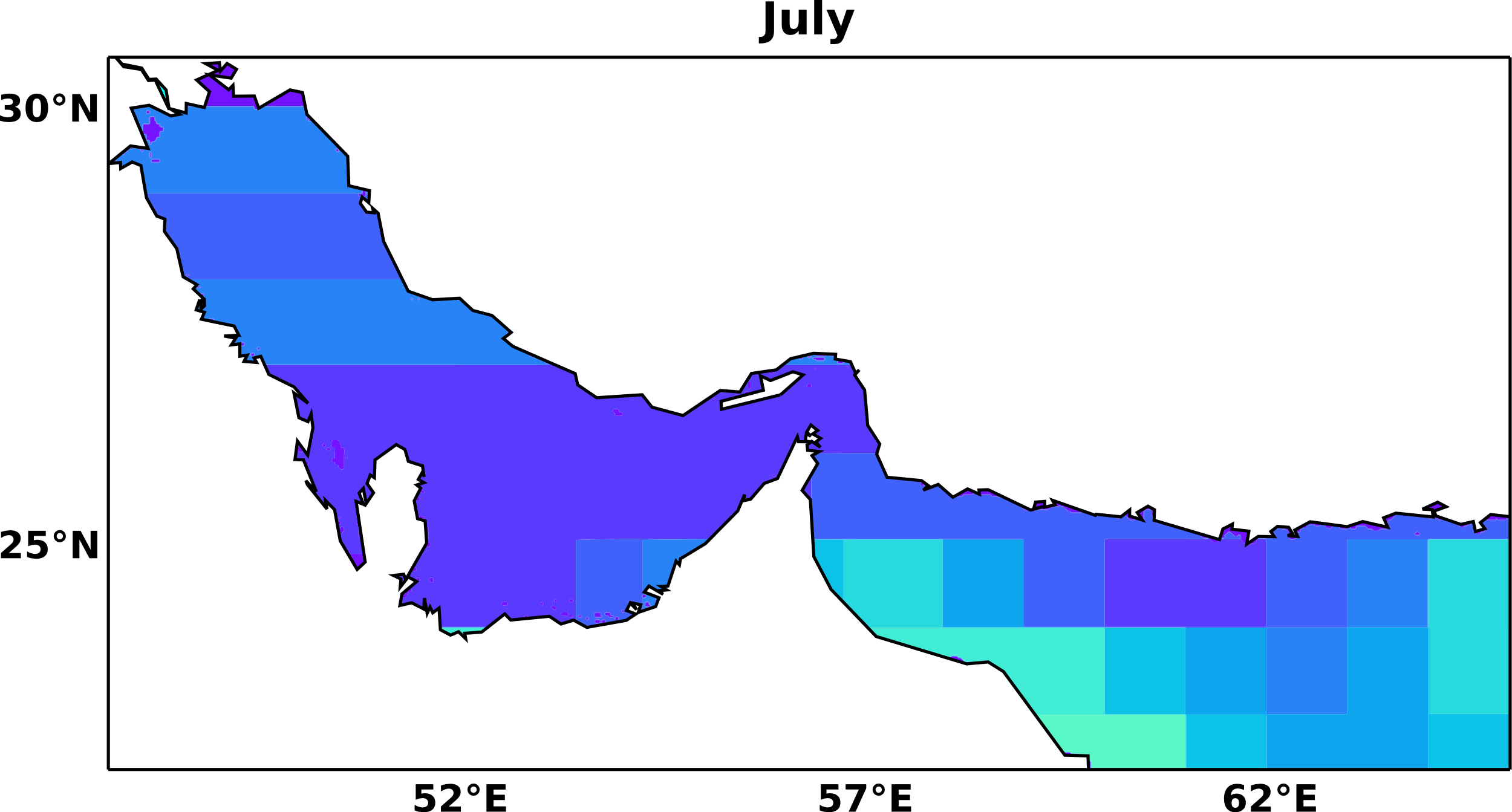

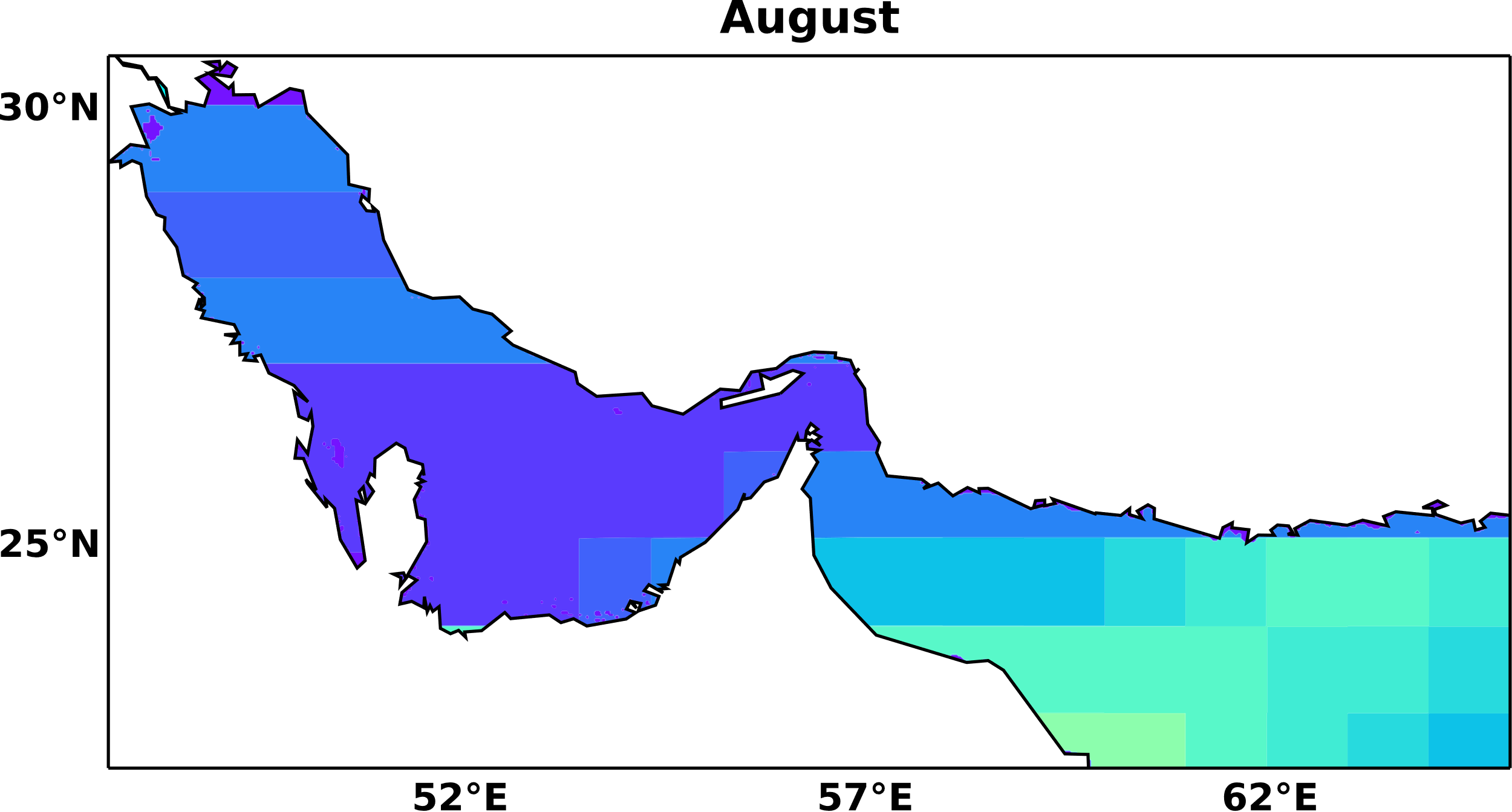

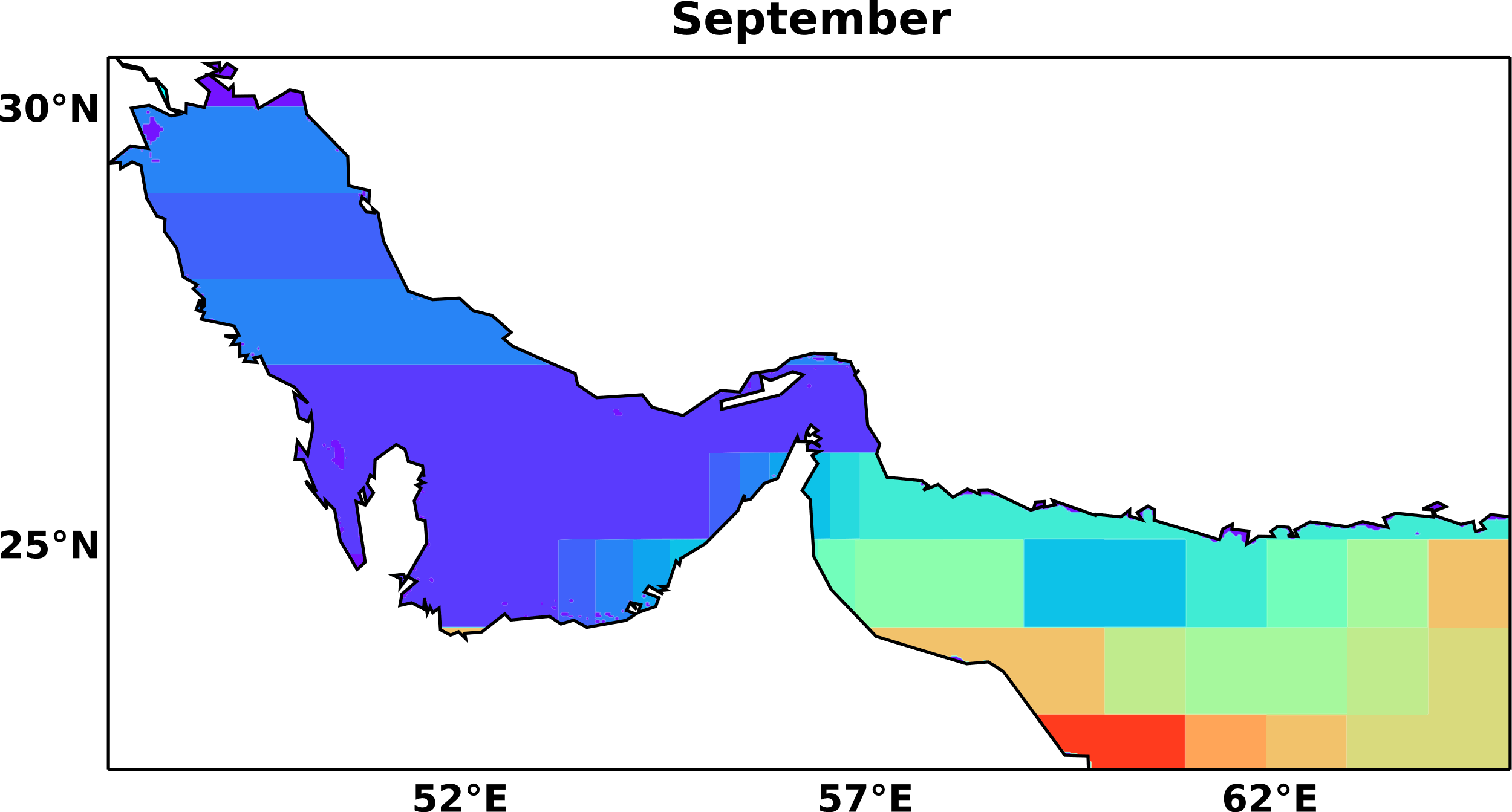

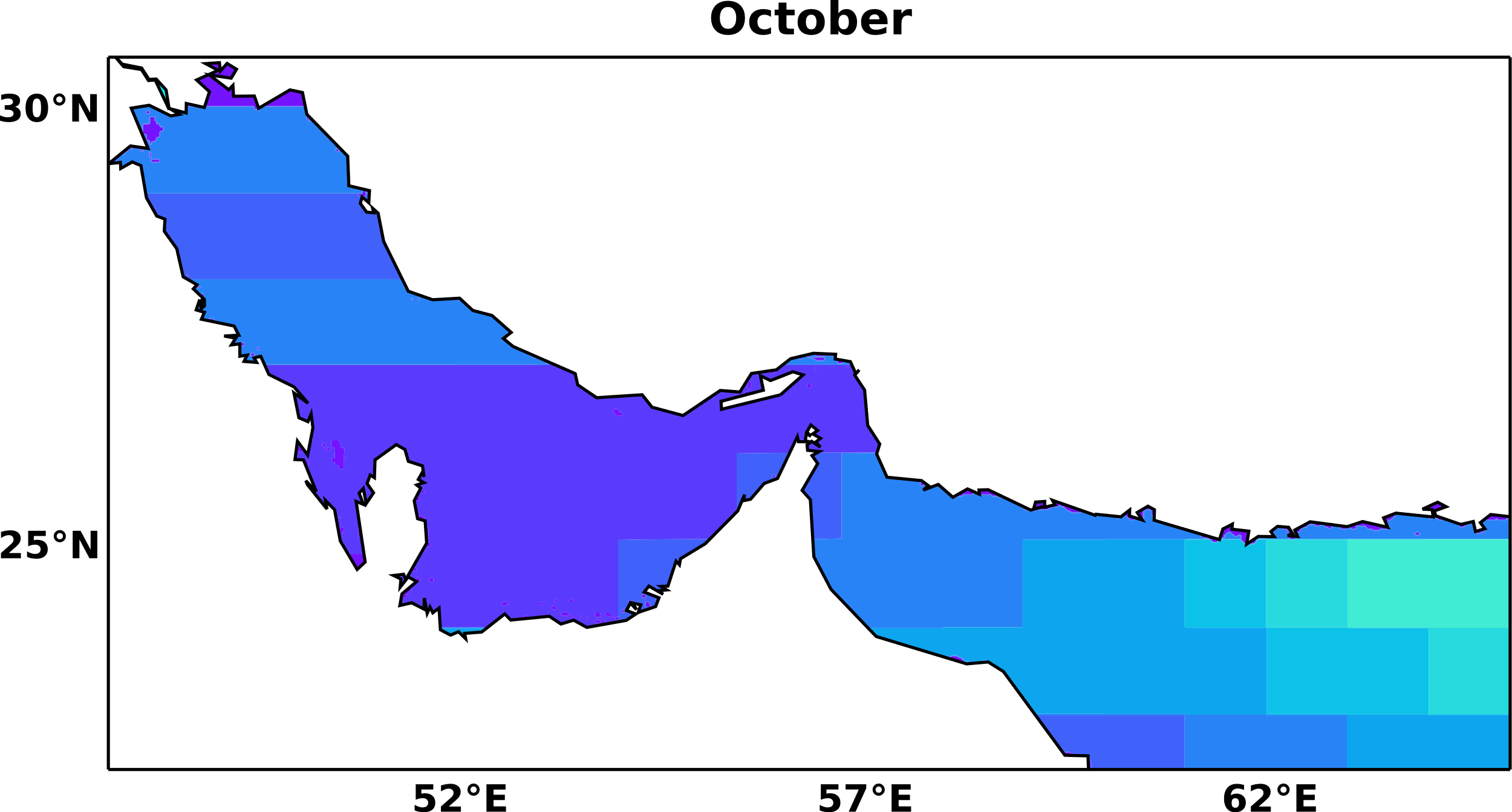

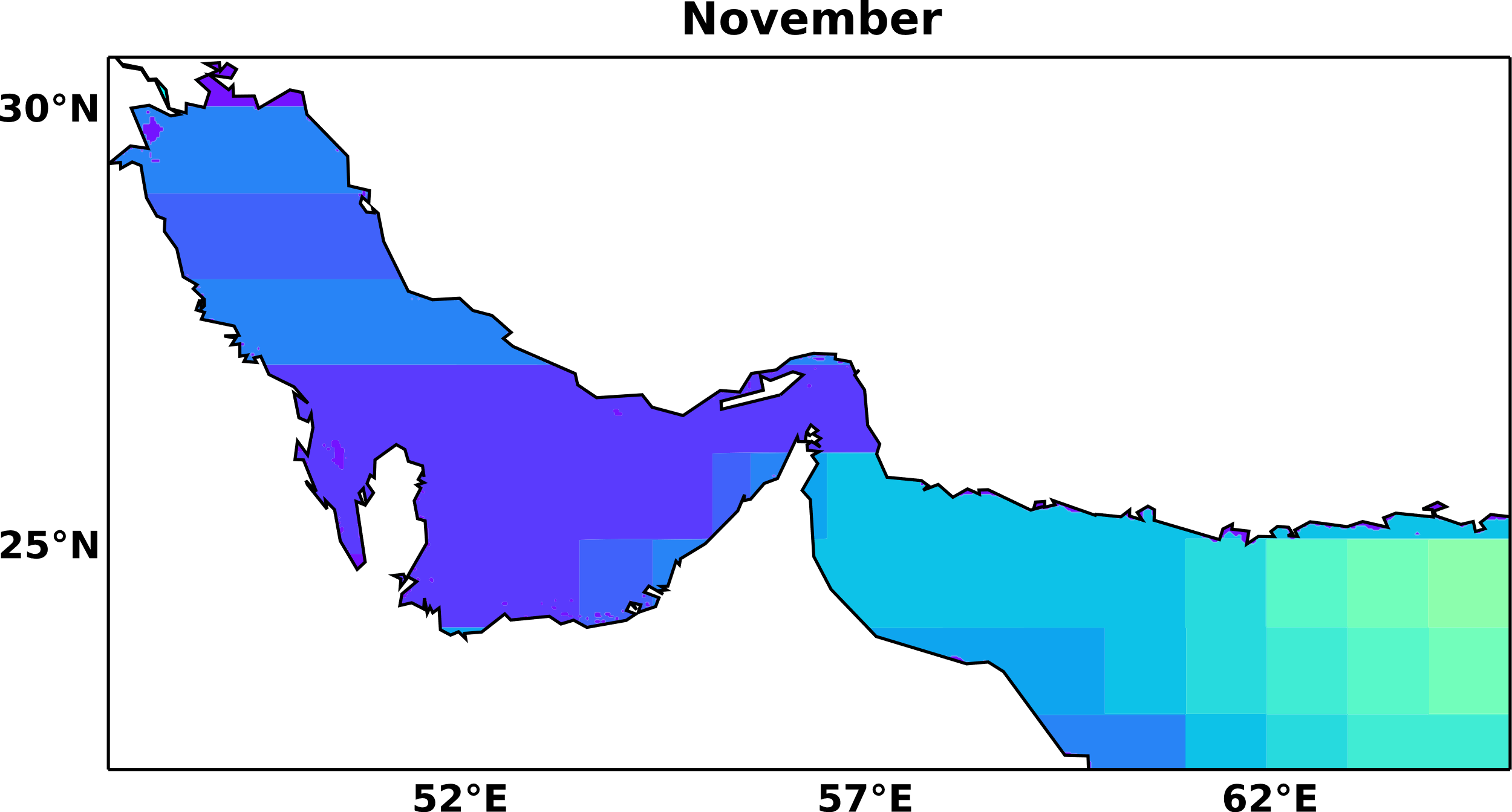

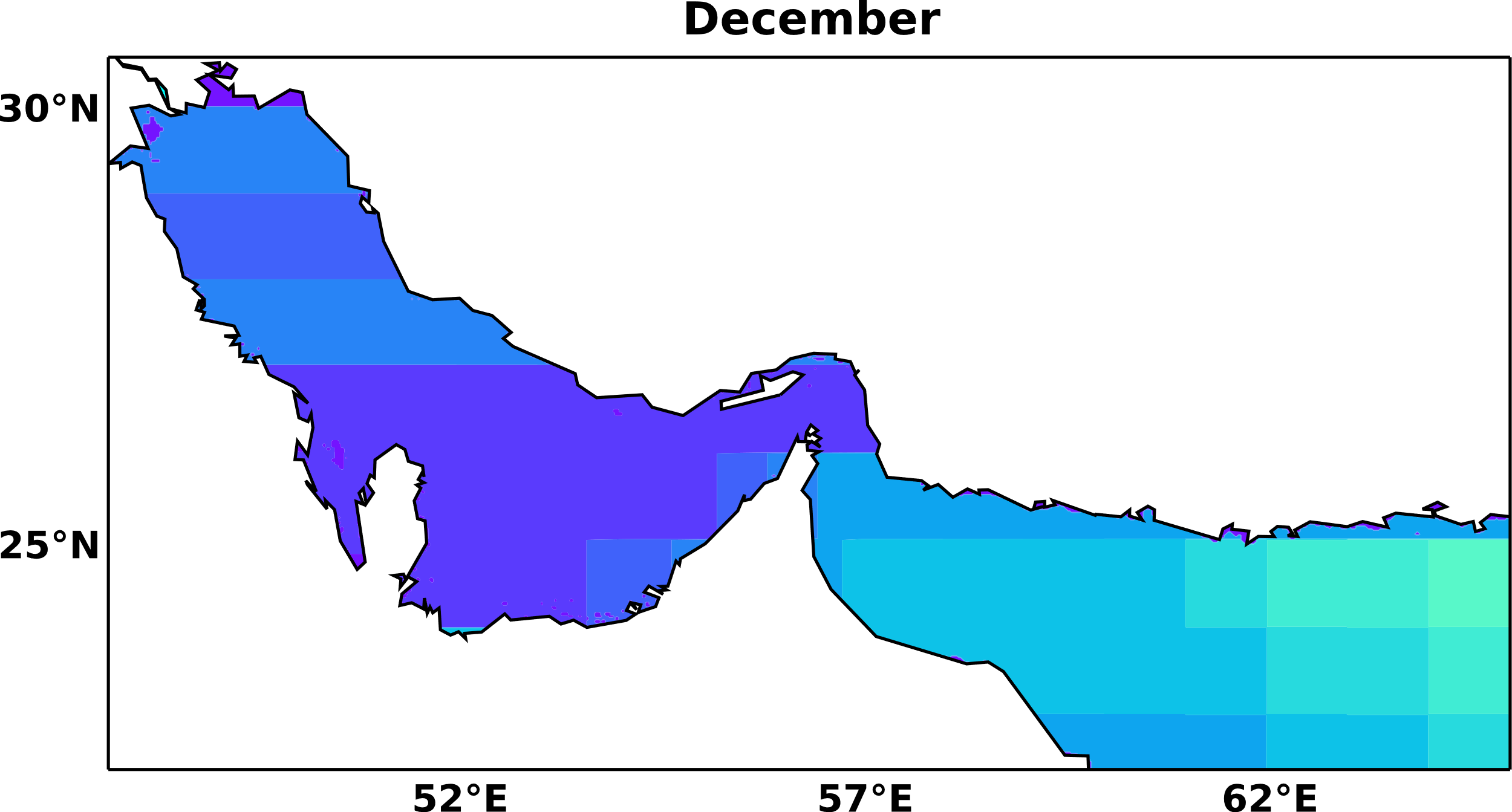

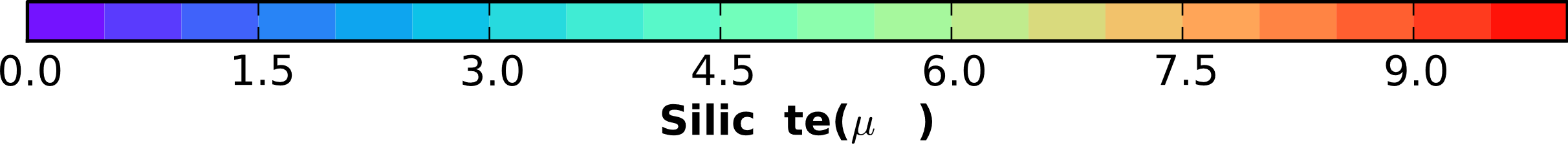


**Silicate (µM)**

**Fig A.2.** Monthly surface silicate climatology for the Arabian Gulf and Sea of Oman extracted from the World Ocean Atlas data (WOA) for the year 2018.


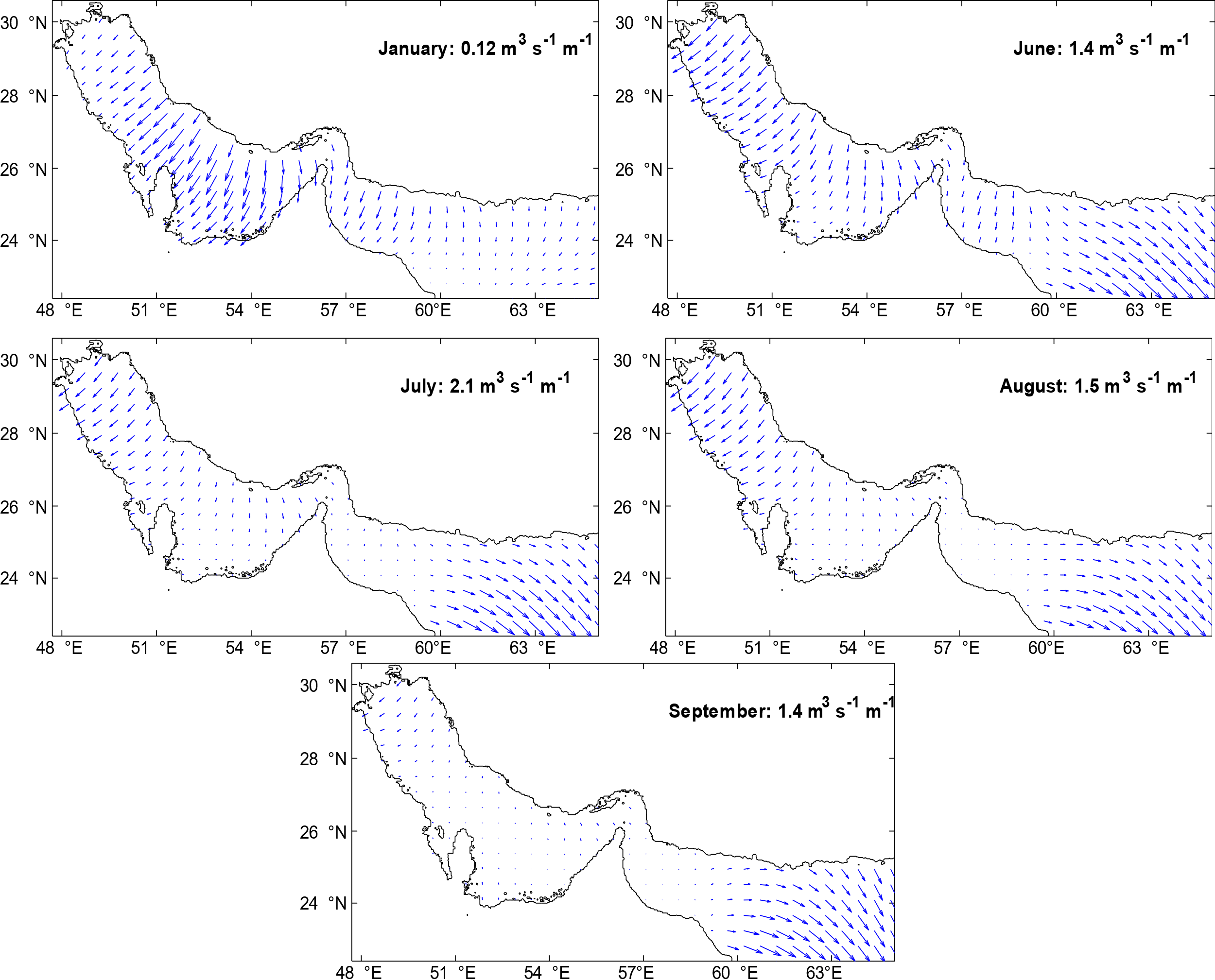


**Fig A.3.** Ekman transport (unit: m^3^ s^-1^ m^-1^) monthly climatology for the months: January, June, July, August, and September where upwelling conditions prevail. Ekman transport is calculated from the wind data extracted from the ECMWF (ERA5) for the year 2018. ERA5 **Climatologies have been calculated over the long-term average period of 1981-2010.** The value on the map represents the highest intensity of the offshore transport which is observed in the Sea of Oman in the months (June, July, August, and September).


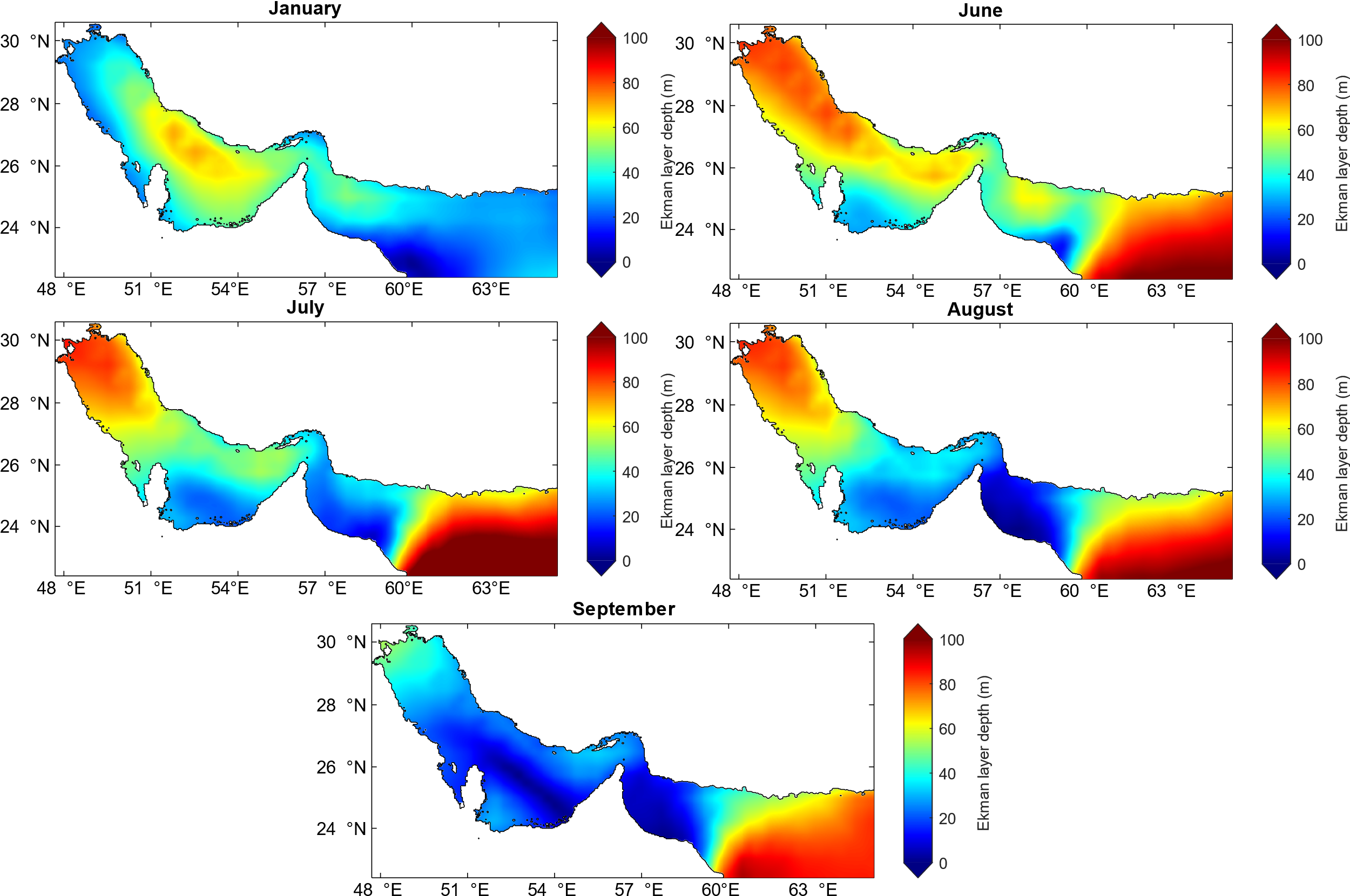


**Fig A.4.** Ekman layer depth (m) for the months (January, June, July, August, September) where the highest upwelling conditions are found over the Arabian Gulf and Sea of Oman. Ekman layer depth is calculated from the wind speed which is extracted from the ECMWF (ERA5) datasets for the monthly climatology of the year 2018. ERA5 **Climatologies have been calculated over the long-term average period of 1981-2010.**


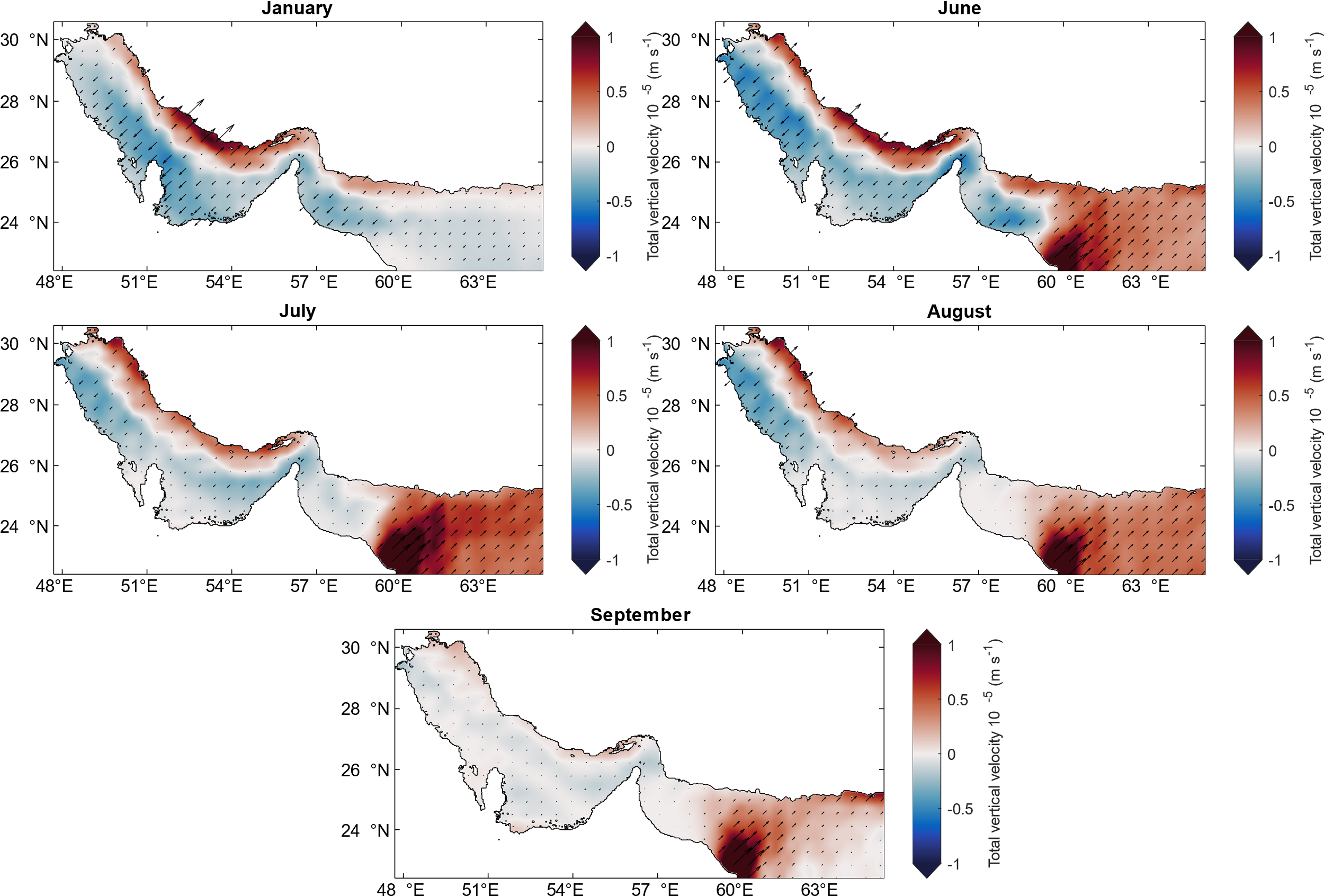


**Fig A.5.** Total vertical velocity (color) due to alongshore wind stress (coastal upwelling) and wind-curl (open sea upwelling) for the months (January, June, July, August, September) where the highest upwelling conditions occur over the Arabian Gulf and Sea of Oman calculated from the monthly climatology of wind speed parameter that is extracted from the ECMWF (ERA5) datasets for the year 2018. ERA5 **Climatologies have been calculated over the long-term average period of 1981-2010.** The vectors on the map show the direction of the velocity which is upward for a positive vertical velocity and downward for a negative vertical velocity.
